# Proceedings of the 19th annual conference of INEBRIA

**DOI:** 10.1186/s13722-023-00431-9

**Published:** 2024-01-30

## Symposia

## NC-S01 Evaluating digital interventions for alcohol and other drugs

### Melissa Oldham^1^, Claire Garnett^1^, Lorien Abroms^2^, Lillian Gelberg^3,4^

#### ^1^Department of Behavioural Science and Health, University College London, UK; ^2^George Washington University, Washington, DC, USA; ^3^Department of Family Medicine, David Geffen School of Medicine at UCLA, Los Angeles, California, USA; ^4^Department of Health Policy & Management, Fielding School of Public Health, UCLA, Los Angeles, California, USA

##### **Correspondence:** Melissa Oldham (m.oldham@ucl.ac.uk)

*Addiction Science & Clinical Practice* 2023, **19(Suppl 1):**NC-S01

**Goal:** Digital interventions can overcome barriers to delivery of face-to-face interventions, as they have the advantage of being remotely available 24/7 and having a broad reach. There is also evidence that digital interventions can have a moderate impact on a variety of health behaviours. A major challenge facing the field of digital interventions is the huge number of interventions that are available and inconsistency in the extent to which interventions are designed with reference to evidence and theory, and the extent to which they are evaluated as being effective and acceptable to users. As such evaluation at different stages of development and implementation is required. In this session, we will hear four talks on the evaluation of digital interventions for alcohol and other drugs at different stages of development and implementation. Dr. Melissa Oldham will present findings from a large Randomised Control Trial (RCT) evaluating the effectiveness of the recommendation of the app, Drink Less, in reducing alcohol consumption amongst increasing and higher risk drinkers in the UK, compared with usual digital care. Dr. Claire Garnett will present the process evaluation of the same trial examining the acceptability of and engagement with the Drink Less app. Professor Lorien Abroms will discuss the feasibility, acceptability and effectiveness of using Electronic Health Records to identify at-risk individuals and using text-messages to signpost them to behaviour change apps for smoking and drinking. Professor Lillian Gelberg will discuss the implementation of a digital Screening, Brief Intervention and Referral to Treatment (SBIRT) for risky drug use among diverse low-income primary care patients.

## Oral presentation

## NC-S01.1 Evaluating the effectiveness of the Drink Less app in reducing consumption amongst increasing and higher risk drinkers in the UK compared with usual digital care

### Melissa Oldham^1^, Emma Beard^1^, Gemma Loebenberg^1^, Larisa Dinu^1^, Colin Angus^2^, Robyn Burton^3,4^, Matt Field^5^, Felix Greaves^6,7^, Matthew Hickman^8^, Eileen Kaner^9^, Susan Michie^10^, Marcus Munafò^11,12^, Elena Pizzo^13^, Jamie Brown^1^, Claire Garnett^1^

#### ^1^Department of Behavioural Science and Health, University College London, UK; ^2^School of Health and Related Research, University of Sheffield, Sheffield, UK; ^3^Addictions Directorate, Office for Health Improvement and Disparities, London, UK; ^4^Institute of Psychiatry, Psychology and Neuroscience, Kings College London, UK; ^5^Department of Psychology, University of Sheffield, Sheffield, UK; ^6^Department of Primary Care and Public Health, Imperial College London, London, UK; ^7^National Institute for Health and Care Excellence, London, UK; ^8^Population Health Sciences, Bristol Medical School, University of Bristol, Bristol; UK; ^9^Population Health Sciences Institute, Newcastle University, Newcastle upon Tyne, UK; ^10^Centre for Behaviour Change, University College London, London, UK; ^11^School of Psychological Science, University of Bristol, Bristol, UK; ^12^MRC Integrative Epidemiology Unit, University of Bristol, Bristol; UK; ^13^Department of Applied Health Research, University College London, London, UK

##### **Correspondence:** Melissa Oldham (m.oldham@ucl.ac.uk)

*Addiction Science & Clinical Practice* 2023, **19(Suppl 1):**NC-S01.1

**Background:** Digital interventions can be effective for reducing alcohol consumption, but many are not evidence- or theory-informed and do not undergo evaluation. The aim of this study was to evaluate the effectiveness of recommending the Drink Less app in reducing alcohol consumption compared with usual digital care.

**Methods:** Two-arm remote randomised controlled trial comparing the effectiveness of recommending the Drink Less app (intervention) compared with the NHS alcohol advice webpage (comparator and usual digital care). 5602 increasing and higher risk drinkers (defined as scoring 8+ on the AUDIT) were recruited between July 2020 and March 2022 and responded to follow-up at 1, 3 and 6 months. The primary outcome was self-reported weekly alcohol consumption at 6 months adjusting for baseline consumption. The trial was powered to detect a difference of 2-units.

**Results:** There were no differences in retention rates at 6-months with retention in both conditions at 80%. The pre-registered primary analysis, using a conservative intention-to-treat approach assuming non-responders were drinking at baseline levels of consumption, found a non-significant 1-unit reduction in weekly alcohol consumption at 6-month follow-up (95% CI − 2.67 to 0.70). A pre-registered Bayes factor calculation of 1.17 suggested that the primary outcome was insensitive to detect the hypothesised 2-unit effect. There were also differences in those responding and not responding to 6-month follow-up in terms of education, occupation and income which indicated that data were not missing completely at random. As such, the pre-registered sensitivity analysis that treated missing data more appropriately, using multiple imputation, showed the Drink Less app resulted in a significant reduction of 2-units at 6-month follow-up compared with usual digital care (95% CI − 3.76 to − 0.24).

**Conclusion:** Recommending the use of the Drink Less app appears effective in helping increasing and higher risk drinkers reduce their alcohol consumption.

## Oral presentation

## NC-S01.2 The acceptability of and engagement with the Drink Less app

### Claire Garnett^1,2^, Larisa Dinu^1^, Melissa Oldham^1^, Gemma Loebenberg^1^, Olga Perski^3,4^, Emma Beard^1^, Colin Angus^5^, Robyn Burton^6,7^, Matt Field^8^, Felix Greaves^9,10^, Matthew Hickman^11^, Eileen Kaner^12^, Susan Michie^13^, Marcus Munafò^2,14^, Elena Pizzo^15^, Jamie Brown^1^

#### ^1^Department of Behavioural Science and Health, University College London, UK; ^2^School of Psychological Science, University of Bristol, Bristol, UK; ^3^University of California, San Diego, USA; ^4^Tampere University, Finland; ^5^School of Health and Related Research, University of Sheffield, Sheffield, UK; ^6^Addictions Directorate, Office for Health Improvement and Disparities, London, UK; ^7^Institute of Psychiatry, Psychology and Neuroscience, Kings College London, UK; ^8^Department of Psychology, University of Sheffield, Sheffield, UK; ^9^Department of Primary Care and Public Health, Imperial College London, London, UK; ^10^National Institute for Health and Care Excellence, London, UK; ^11^Population Health Sciences, Bristol Medical School, University of Bristol, Bristol, UK; ^12^Population Health Sciences Institute, Newcastle University, Newcastle upon Tyne, UK; ^13^Centre for Behaviour Change, University College London, London, UK; ^14^MRC Integrative Epidemiology Unit, University of Bristol, Bristol, UK; ^15^Department of Applied Health Research, University College London, London, UK

##### **Correspondence:** Claire Garnett (claire.garnett@bristol.ac.uk)

*Addiction Science & Clinical Practice* 2023, **19(Suppl 1):**NC-S01.2

**Background:** Perceived acceptability and engagement are necessary for interventions to be effective and widely used. Drink Less is a popular, evidence- and theory-informed alcohol reduction app in the UK. We aimed to investigate the engagement with and acceptability of Drink Less among increasing and higher risk drinkers as part of a process evaluation embedded within a larger randomised controlled trial (n = 5602).

**Methods:** We conducted a mixed-methods evaluation to assess engagement and acceptability. Engagement with Drink Less among participants in the intervention group (n = 2788) was collected automatically via the app and assessed in terms of app download, and frequency, amount, duration, and depth of engagement over six months from app download. The acceptability of the interventions were assessed in a number of short semi-structured interviews among a sub-sample of participants (n = 26) after the six-month follow-up, according to the Theoretical Framework of Acceptability.

**Results:** Participants had a mean of 42 sessions (frequency, SD = 66.51) on Drink Less and spent a mean of 1 h 9 min on it (amount, SD = 2h3m). Participants viewed a mean of 23.6 different screens (depth, SD = 10.41) and spent a mean of 30 days when they used the app (duration, SD = 43.94). Participants rated the intervention highly in terms of its acceptability; they reported liking the tool, felt it was effective and accessible, quick and easy to use, and reported trusting the app because of the association with University College London. Over two-thirds (67.0%, n = 1869) of participants downloaded and used Drink Less when recommended it in the intervention group.

**Conclusions:** Increasing and higher risk drinkers engaged frequently with the Drink Less app and perceived the intervention to be acceptable.

## Oral presentation

## NC-S01.4 Implementing screening, brief intervention, and referral to treatment (SBIRT) for risky drug use in FQHC primary care clinics in the COVID-19 era: the QUIT-Mobile study

### Lillian Gelberg^1,2^, Stephanie Sumstine-Felice^3^, Leticia Cazares^1^, Quynh Vo^1^, Cristina Hernandez^1^, Cristina Batarse^1^, Daisy Hernandez-Casas^1^, Melvin W. Rico^1,4^, Dallas Swendeman^3^

#### ^1^Department of Family Medicine, David Geffen School of Medicine at UCLA, Los Angeles, California, USA; ^2^Department of Health Policy & Management, Fielding School of Public Health, UCLA, Los Angeles, California, USA; ^3^Department of Psychiatry and Biobehavioral Sciences, David Geffen School of Medicine at UCLA, Los Angeles, California, USA; ^4^Charles R. Drew University of Medicine and Science, Los Angeles, California, USA

##### **Correspondence:** Lillian Gelberg (lgelberg@mednet.ucla.edu)

*Addiction Science & Clinical Practice* 2023, **19(Suppl 1):**NC-S01.4

**Background:** The Quit Using Drugs Intervention Trial Mobile (QUIT-Mobile) study is a NIH/NIDA-funded Hybrid Type-1 effectiveness-implementation randomized controlled trial of screening and brief intervention (SBI) aimed at reducing moderate risk drug use (ASSIST score 4–26; ASAM level 0.5) among diverse low-income primary care patients in Los Angeles. Adaptation factors for optimal uptake and sustainability during COVID-19 and telehealth expansion are assessed.

**Methods:** Meetings with clinic partner stakeholders (chief medical officers, doctors, IT managers, health education managers, research coordinators) address barriers and facilitators to adoption and implementation. Two multi-clinic early adopters participated in piloting and initial implementation while several others declined. Thematic analysis of meeting notes is guided by the Consolidated Framework for Implementation Research (CFIR).

**Results:** Key themes include: (1) perceived advantage implementing QUIT-Mobile given lack of SBIs and increased drug use during COVID-19; (2) flexibility adapting protocols to not disrupt clinic flow and to meet clinic compliance standards; (3) trialability piloting with quality improvement PDSA cycles given staff burnout, turnover and capacity limitations, and shifting in-person to telehealth patient-ratios; (4) costs of implementation and sustainment addressed by identifying reimbursement opportunities; (5) meeting patient needs to alleviate mistrust and develop effective referral protocols; (6) relative priority fostering collaborative implementation climate and utilizing mobile-web patient self-administered pre-visit screener and consent; (7) clinician champions keeping staff and providers engaged and updated, and problem solving challenges with the study team.

**Conclusion:** Findings demonstrate challenges in telehealth changes while navigating clinic challenges. Next steps include assessing barriers and facilitators to implementation after study launch and completion. If effective, this SBI will be integrated into routine primary care following recommendations of the Affordable Care and Mental Health Parity Acts.

## NC-S02 Findings from a two-arm parallel group individually randomised prison pilot study of a male remand alcohol intervention for self-efficacy enhancement: the APPRAISE study

### Aisha Holloway

#### Nursing Studies, School of Health in Social Sciences, University of Edinburgh, Scotland, UK

##### **Correspondence:** Aisha Holloway (aisha.holloway@ed.ac.uk)

*Addiction Science & Clinical Practice* 2023, **19(Suppl 1):**NC-S02

**Goal:** As many as 70% of remand prisoners have admitted to being under the influence of alcohol when committing the crime leading to their imprisonment. Providing support and advice regarding alcohol consumption can be effective in some groups of people. There is little evidence regarding this for men on remand in prison. The aim of the symposium is to present the key findings from a two-arm, parallel group, individually randomised pilot study of a self-efficacy enhancing psychosocial alcohol intervention, the APPRAISE study, to reduce levels of alcohol consumption for males on remand in prison and on liberation conducted in the United Kingdom. The goal of the symposium are to present four key sub-studies of the APPRAISE study:

1. Pilot trial to pilot the study measures and evaluation methods to assess the feasibility of conducting a future definitive multi-centre, pragmatic, parallel group, RCT.
2. Embedded process evaluation to assess intervention fidelity and explore the feasibility and acceptability of a self-efficacy enhancing psychosocial alcohol intervention and study measures to staff and for men on remand and on liberation.
3. Economic evaluation pilot study to ascertain if we could collect economic data needed for a future definitive RCT; access recidivism data from the Police National Computer (PNC) databases for trial participants; and access health data from routine NHS data sources for trial participants.
4. Alcohol service survey of what alcohol services in prisons across England, Wales and Scotland are available in male remand prisons and how COVID-19 affected.

## Oral presentation

## NC-S02.1 Pilot trial to pilot the study measures and evaluation methods to assess the feasibility of conducting a future definitive multi-centre, pragmatic, parallel group, RCT: the APPRAISE study

### Aisha S. Holloway

#### Nursing Studies, School of Health in Social Sciences, University of Edinburgh, Scotland, UK

##### **Correspondence:** Aisha Holloway (aisha.holloway@ed.ac.uk)

*Addiction Science & Clinical Practice* 2023, **19(Suppl 1):**NC-S02.1

**Background:** Support and advice regarding alcohol consumption can be effective however, there is little evidence regarding this for men on remand in prison. We conducted a two-arm, parallel group, individually randomised pilot study of a self-efficacy enhancing psychosocial alcohol intervention to reduce levels of alcohol consumption for males on remand in prison and on liberation.

**Materials and methods:** Two purposively selected prisons in Scotland and England. Adult men on remand in prison with an Alcohol Use Disorders Identification Test score of ≥ 8 were consented and recruited to the study and randomised to intervention or control. Recruitment target was 180, 90 at each study site. The APPRAISE intervention comprised 1 × 40-min face-to-face session in prison and 4 × 20-min sessions conducted by phone, on or as close to day 3, 7, and 21 post liberation. Appropriate ethical approval and participant consent was obtained.

**Results:** Of 182 men on remand approached across two study sites, 132 were randomised [90 in England; 42 in Scotland] with 46 randomised to intervention and 44 to care as usual in England; 22 randomised to intervention and 20 to care as usual in Scotland. A total of 53 in prison interventions were delivered. 1 day 3 post liberation intervention delivered, no day 7 and 1 day 21. At 12 months, of 132 randomised, 18 (13%) were followed up, 53 (40%) were not liberated; 47 (36%) were uncontactable and 14 (11%) were released but unable to be located. Data completeness was 96% at baseline and 8% at 12 months. Although the sample sizes are small, exploratory results suggest that self-efficacy is an important determinant of alcohol consumption and interventions should primarily target self-efficacy.

**Conclusions:** A future definitive trial would be possible, but only if follow-up mechanisms can be addressed.

## Oral presentation

## NC-S02.2 Assessing intervention fidelity and exploring the feasibility and acceptability of a self-efficacy enhancing psychosocial alcohol intervention and study measures to staff and for men on remand and on liberation

### Aisha S. Holloway

#### Nursing Studies, School of Health in Social Sciences, University of Edinburgh, Scotland, UK

##### **Correspondence:** Aisha Holloway (aisha.holloway@ed.ac.uk)

*Addiction Science & Clinical Practice* 2023, **19(Suppl 1):**NC-S02.2

**Background:** An embedded process evaluation, as part of the APPRAISE study is a two-arm, parallel group, individually randomised pilot study of a self-efficacy enhancing psychosocial alcohol intervention to reduce levels of alcohol consumption for men on remand in prison and on liberation was conducted to ascertain intervention fidelity, feasibility and acceptability.

**Materials and methods:** Fifteen semi-structured interviews were conducted with three participant groups from across the two study sites: men on remand participating in APPRAISE study; those delivering the intervention and prison personnel and study researchers. The Behaviour Change Counselling Index (BECCI), length of time for intervention delivery and number of interventions delivered provided data for the TiDieR Checklist. The qualitative analyses was undertaken through the lens of Normalisation Process Theory (NPT). Appropriate ethical approval and patient consent was obtained.

**Results:** Findings from qualitative interviews with men who received the intervention and those delivering it demonstrated that the APPRAISE intervention in-prison ASBI sessions were acceptable, appropriate and welcomed. Post-release sessions were not feasible mainly due to participants not being contactable and the impact of Covid-19. The Behaviour Change Counselling Index (BECCI) scores identified that the 4 recorded intervention delivery sessions were delivered as per protocol.

**Conclusion:** The prison setting and culture affected the acceptability and feasibility of the ABI. The ABI delivery benefited from staff buying into the intervention and being motivated and engaged. The stakeholder data identified the importance of engagement with prisoners and the lack of currently available support for males on remand. Overall, the process evaluation reported good acceptability of the intervention with investment in time, capacity and space to support implementation identified. Future work should focus on determining the feasibility and acceptability of the ABI on a wider scale and addressing feasibility of post release intervention delivery outside of a pandemic.

## Oral presentation

## NC-S02.3 Implementation costs of the APPRAISE alcohol brief intervention (ABI) for male remand prisoners: a micro-costing protocol and preliminary findings

### Gillian Waller^1^, Jennifer Ferguson^1^, Jeremy W. Bray^2^, Dorothy Newbury-Birch^1^, Andrew Stoddart^3^, Aisha Holloway^4^

#### ^1^School of Social Sciences, Humanities and Law, Teesside University, Middlesbrough, England, UK; ^2^UNC Greensboro, Greensboro, NC, USA; ^3^Edinburgh Clinical Trials Unit (ECTU), University of Edinburgh, Scotland, UK; ^4^Nursing Studies, School of Health in Social Sciences, University of Edinburgh, Scotland, UK

##### **Correspondence:** Jeremy W. Bray (jwbray@uncg.edu)

*Addiction Science & Clinical Practice* 2023, **19(Suppl 1):**NC-S02.3

**Background:** This paper documents the methods used to assess the implementation costs of the APPRAISE intervention, an alcohol brief intervention (ABI) delivered to male remand prisoners across two study sites in Scotland and Northern England, in comparison to usual care.

**Materials and methods:** As a subset of the wider APPRAISE pilot; this embedded work sought to assess the costs associated with delivering the ABI. It also sought to develop a methodology for estimating downstream health and social sector costs. We first developed a comprehensive taxonomy of the activities constituting the APPRIASE ABI. Next, data were collected for each activity about the study staff and the subject time spent, in addition to the other resources used and unit costs.

**Results:** From the pilot data collection, it was possible to construct a narrative, for both study sites, for how prisoner recruitment took place and the time required for each activity. The ABI was delivered by Change Grow Live and Humankind intervention staff and hence staff salaries were obtained from both organisations to calculate the staff delivery costs for each site. Other costs, such as the printing of materials, were estimated based on APPRAISE study records. Due to ongoing Covid-19 restrictions and limited access to prison resources and staff, there were significant deviations from the initial study protocols. As a result, we document the costs if implementing the ABI as delivered rather than as planned.

**Conclusions:** This paper provides the first estimates of the implementation costs of an ABI delivered in criminal justice setting. Although these costs are from a pilot implementation heavily impacted by the Covid-19 pandemic, this paper nonetheless provides useful, policy-relevant information on the potential costs of providing ABI to remand prisoners. It also serves as a methodological template and guidance for future micro-costing studies of ABIs in criminal justice settings.

## Oral presentation

## NC-S02.4 Survey of male remand prisons in England, Scotland, and Wales: the APPRAISE study

### Jennifer Ferguson

#### Teesside University, Middlesbrough, England, UK

##### **Correspondence:** Jennifer Ferguson (jennifer.ferguson@tees.ac.uk)

*Addiction Science & Clinical Practice* 2023, **19(Suppl 1):**NC-S02.4

**Background:** The COVID-19 pandemic affected every aspect of society and prisons across the world immediately went into lockdown. This left prisoners being unable to maintain contact with anyone, and they often found themselves isolated and confined to their cells for extended periods of time, in order to minimize the risk of a COVID-19 outbreak. We anticipated by undertaking this survey it would help us to understand what the prisons did in relation to alcohol services throughout the pandemic, and filling in the gaps in our data that the research team were not able to complete as part of APPRAISE.

**Methods and materials:** An online survey was distributed to 55 prison governors in England, Wales and Scotland by email invitation. A total of 17 prisons completed the survey. SPSS was employed to analyse the data.

**Results:** We were able to ascertain what knowledge of alcohol brief interventions is held by prison governors and what alcohol services are currently being delivered. The survey showed that a broad range of services are being delivered, by a range of service providers. However, it was also clear that the COVID-19 pandemic had a devastating affect across all prisons in relation to the services they provide. It was also useful to establish what the prison governors deemed important learning from the pandemic, in this instance mostly around PPE and a better use of technology.

**Conclusions:** Prisons services for alcohol were affected by the Covid-19 pandemic and we now have a clearer understanding of how the wider APPRAISE study was affected.

## NC-S03 Optimizing implementation of alcohol screening and brief interventions through digital approaches

### Joan Colom

#### Subdirectorate General on Addictions, HIV, STD and Viral Hepatitis, Public Health Agency of Catalonia, Barcelona, Catalonia, Spain

##### **Correspondence:** Joan Colom (joan.colom@gencat.cat)

*Addiction Science & Clinical Practice* 2023, **19(Suppl 1):**NC-S03

**Goal:** Alcohol screening and brief interventions (aSBI) can be delivered via multiple digital modalities, and the efficacy of digital aSBI does not seem to significantly vary depending on the type of device, with most studies indicating a reduction in drinking or alcohol-related harm. Digital approaches have a great potential to adapt interventions to the needs of diverse population groups, including less advantaged people by presenting, for instance, bite-sized information in plain language, accompanied by visuals, animations, reading functions. Based on available evidence supporting the efficacy of digital aSBI, and given the increasing levels of digital usage and literacy in the general population, digital alcohol interventions could be a low threshold and low cost option in primary healthcare, particularly in low-resource environments. Digital aSBI may contribute to increasing the generally low rates of professional-delivered alcohol use assessment and brief advice, and reduce workload and time-pressure for health professionals, thus release resources for patients who require complex management. However, despite the large pool of evidence, scalability from pilot studies to large-scale implementation remains an issue, and makes the evaluation of effectiveness in real-life circumstances difficult. Despite the general push for a digital revolution in service delivery and the advance of many small-scale proof-of-concept examples, digital aSBI is rarely mainstreamed or sustained in the health system. The symposium aims to shed light on some of the critical topics and lessons learned in relation to large-scale implementation approaches of evidence-based digital alcohol interventions, including health, socioeconomic and digital inequalities; provider-facilitated approaches; self-initiated use; and emerging digital trends in health and alcohol literacy acquisition, and digitally driven provider/user interactions.

## Oral presentation

## NC-S03.1 Evidence and considerations around health and socioeconomic inequalities: who benefits from digital alcohol interventions?

### Silvia Matrai, Hugo Lopez Pelayo, Antoni Gual

#### Health and Addictions Research Group, CLÍNIC Foundation-IDIBAPS, Barcelona, Catalonia, Spain

##### **Correspondence:** Silvia Matrai (smatrai@recerca.clinic.cat)

*Addiction Science & Clinical Practice* 2023, **19(Suppl 1):**NC-S03.1

**Introduction:** There is a steady evidence base for digital alcohol interventions in general populations, but there is scarce evidence in population groups that might be expected to be excluded, such as the elderly, people from low socioeconomic background, and ethnic minorities. While transition towards digital approaches in health promotion and care is becoming increasingly normative, most digital health designs and research have been made in advantaged populations, with limited or no participation of population groups at risk of socioeconomic, health and digital inequalities.

**Methods:** Within the EU-funded AlHaMBRA Project, we conducted a literature review of the current knowledge of digital alcohol prevention and treatment approaches in vulnerable populations groups including people from low socioeconomic status, migrants, young people, and women of child-bearing age. We also reviewed evidence of the unintended effects of such approaches in terms of social and health inequalities.

**Results:** In general, digital approaches have a great potential to adapt interventions to the needs of less advantaged people, for instance, by presenting bite-sized information in plain language, accompanied by visuals, animations, reading functions, and speech recognition. Key requirements for engagement in digital alcohol tools in young people include professional and text-light design, interactive and audio-visual components, and credited information source. Digital approaches may be particularly suitable to help women of childbearing age feel less embarrassment and fear of judgement about their drinking.

**Conclusions:** Population groups who are less likely to access or turn to usual alcohol-related services might experience the greatest benefit from using digital technologies to prevent and treat alcohol consumption. In order to maximise the acceptability and usability of digital alcohol approaches in vulnerable groups, the principles of equitable design, an intersectional approach and co-creation techniques should be used to address critical social, economic, cultural, and health factors when ideating, developing, testing and implementing such approaches.

## Oral presentation

## NC-S03.2 Digital innovation in healthcare: lessons learned from implementation of provider-facilitated digital alcohol SBIRT in PHC

### Lidia Segura-Garcia^1^, Carla Bruguera^1^, Silvia Matrai^2^, Joan Colom^1^, Hugo Lopez-Pelayo^2^, Antoni Gual^2^

#### ^1^Subdirectorate General on Addictions, HIV, STD and Viral Hepatitis, Public Health Agency of Catalonia, Barcelona, Catalonia, Spain; ^2^Health and Addictions Research Group, CLÍNIC Foundation-IDIBAPS, Barcelona, Catalonia, Spain

##### **Correspondence:** Lidia Segura-Garcia (lidia.segura@gencat.cat)

*Addiction Science & Clinical Practice* 2023, **19(Suppl 1):**NC-S03.2

**Introduction:** Within the Beveu Menys, aimed at disseminating throughout PHC the strategies for alcohol screening, brief intervention and referral to treatment, we tested the effectiveness of integrating the provider facilitated access to an electronic aSBI tool in two different studies.

**Methods:** (1) ODHIN was a multicentric study trial involving 120 PH centers from 5 different countries. (2) EFAR was a randomized non-inferiority controlled trial that recruited 115 healthcare professionals.

**Results:** Odhin found that the use of eSBI did not result in any increase in the proportion of patients screened. However, it was associated with an increase in the proportion of screen positive patients receiving brief advice from 70 to 80% for the screen-positive sample. EFAR showed that digital SBI was not inferior to face-to-face BI. However, the low power of the final sample, due to the low recruitment and loss to follow-up (in average, only 2 out of 10 patients logged on to eSBI website) limited the interpretation of the findings. In general, even though professionals valued the digital solutions they also complaint about technical and organizational difficulties that affected the quality of care delivered. In both studies professionals were, in general, not enthusiastic about facilitating access to digital aSBI tools since they did not reduce their time constraints, did not help them in the follow-up of the patients due to the lack of feedback and introduced inequities with elderly and low-socioeconomic population groups. Possible explanations of this low engagement were the age of the majority of the primary healthcare users and low access to the internet.

**Conclusions:** Usefulness perception is a key factor, therefore co-creation process with professionals and general population might lead to a better understanding about how to design effective and easily implementable e-health solutions.

## Oral presentation

## NC-S03.3 Empowering healthcare users: strengthening aSBIRT in primary care through a self-initiated digital tool

### Carla Bruguera^1^, Silvia Matrai^2^, Lidia Segura-Garcia^1^, Joan Colom^1^, Hugo Lopez-Pelayo^2^, Antoni Gual^2^

#### ^1^Subdirectorate General on Addictions, HIV, STD and Viral Hepatitis, Public Health Agency of Catalonia, Barcelona, Catalonia, Spain; ^2^Health and Addictions Research Group, CLÍNIC Foundation-IDIBAPS, Barcelona, Catalonia, Spain

##### **Correspondence:** Carla Bruguera (cbrugueras@gencat.cat)

*Addiction Science & Clinical Practice* 2023, **19(Suppl 1):**NC-S03.3

**Introduction:** Digital alcohol interventions can be facilitated by a health care provider, but also made available for self-initiation and self-management. Anonymous digital alcohol SBI has been found both feasible and acceptable. People with alcohol use problems seem more likely to engage in a digital alcohol intervention through self-initiation. Thus, digital opportunities for self-screening and self-referral may facilitate treatment seeking for problem drinking.

**Methods:** A two-phase pilot study of a patient-initiated online alcohol SBI tool has been implemented in 16 PHC of Catalonia classified according to size (large/small), location (urban/rural) and socioeconomic (low/high). The tool consists of a self-screening of alcohol consumption using AUDIT, estimation of the patient’s alcohol risk profile using self-reported health data, personalised BI, and referral to a digital self-management tool or to follow-up in primary care or specialised settings. The study aims to provide evidence on the utility and feasibility of a large-scale patient-initiated digital approach in different contexts.

**Results:** Preliminary data from Phase 1 of the pilot deployed in eight primary care centres show that, out of the 652 respondents who completed all sections of the digital alcohol SBI tool: 32% completed upper secondary education, and 59% are tertiary graduates; 11% drinks at risky levels, 8% at harmful levels, and 2% may have alcohol dependence, with no significant difference by gender; 41% live in rural areas, and 57% in urban areas. The estimated drop-out rate before data cleansing, i.e. respondents who abandon the tool at any stage before completing, is over 50%. Further results will be prepared for the conference.

**Conclusions:** Phase 1 has led to lessons learned about critical organisational and technological aspects of system-level digital innovation. Data seem to support evidence on the limited uptake of digital alcohol tools in lower educated people, the preference of the respondents to remain anonymous, and the generally high drop-out.

## Oral presentation

## NC-S03.4 The use of digital conversational agents for alcohol education and brief interventions aimed at the general public

### Maristela G. Monteiro

#### International consultant, Pan American Health Organization (retired), Washington DC, USA

##### **Correspondence:** Maristela G. Monteiro (mrstl.monteiro@gmail.com)

*Addiction Science & Clinical Practice* 2023, **19(Suppl 1):**NC-S03.4

**Introduction:** Chatbots and conversational agents became an important piece of the WHO response during the COVID-19 pandemic to quickly disseminate evidence-based information on COVID-19 and tobacco. PAHO seized the opportunity to develop a conversational agent to talk about alcohol related topics, assess people’s risks using AUDIT, and provide a BI to those willing to reduce their drinking.

**Methods:** PAHO secured a non-exclusive worldwide license with a technology company to use their Human OS ecosystem, which enables human-like interactions between digital people and human users via an application, through audiovisual and text interactions. Google Digital flow ES was used to develop the conversations on alcohol and health topics, screening using AUDIT, and providing a quit/cut back plan to users. A communication campaign was implemented from launching date until 31 December 2021.

**Results:** The chatbot, named Pahola, was deployed in August 2021 and launched publicly via a webinar on 19 November 2021. Pahola speaks in English, Spanish and Portuguese, interacts anonymously to a potential infinite number of users through various digital devices. Pahola potentially reached 1.6 million people, leading to 236,000 sessions on its landing page, mostly through mobile devices in the first 2,5 months. However, only 1532 users had an effective conversation with Pahola. The average time people spent talking to Pahola was five minutes; more users were interested in knowing their risk than learning about alcohol topics. Major dropouts were observed in different steps of the conversation flow.

**Conclusions:** Pahola was quickly able to connect to a worldwide population. However, improvements are needed to deal with the public’s trust and use of their devices, ethical issues, integration into health systems and sustainability. The potential of chatbots to educate on alcohol related topics and to increase coverage of SBI seems enormous, but requires a long-term investment of resources and further research.

## Workshops

## NC-W01 Designing electronic screening and brief intervention (e-SBI) applications without coding using the Computerized Intervention Authoring System v. 3.0

### Steven J. Ondersma^1^, Amy M. Loree^2^, Jordan M. Braciszewski^2^

#### ^1^Michigan State University, Flint, Michigan, USA; ^2^Henry Ford Health, Detroit, Michigan, USA

##### **Correspondence:** Steven J. Ondersma (onders12@msu.edu)

*Addiction Science & Clinical Practice* 2023, **19(Suppl 1):**NC-W01

**Description:** Developing digital screening and brief intervention applications is expensive, slow, and requires technical skills as well as content expertise. Further, once developed, custom applications can be difficult to edit, share, adapt, or maintain without significant additional resources. These obstacles impede digital intervention science and stifle innovation. The Computerized Intervention Authoring System (CIAS; www.cias.app) v. 3.0 is an NIH-funded, open-source, non-commercial platform that enables researchers to easily develop, edit, and share sophisticated interactive content without coding of any kind–and with no barriers related to cost. Interventions built with CIAS deploy as cross-platform compatible web/mobile web apps of any duration. Key features include (1) an intuitive interface built following user-centered design principles; (2) optional narrators with many engaging animations who can speak aloud in 45 different languages; (3) instant language translation; (4) motivation-consistent capabilities such as synchronous interactivity, natural language reflections, and formula-driven branching logic; (5) easy incorporation of logos, images, graphs, figures, text, or videos; (6) integrated tailored text messaging; (7) multiple screen templates (single choice, multiple choice, normed feedback, narrator only, etc.); (8) custom summary report generation; (9) aggregate data visualization; (10) integrated secure live chat; and (11) compliance with HIPAA and WCAG 2.0 accessibility standards. Additional capabilities are continually being added in response to input from the entire CIAS ecosystem of researchers, participants, healthcare providers, and other stakeholders.

This workshop will proceed in four phases. First, we will provide an overview of options for digital intervention development, highlighting the advantages of mobile web apps and no-code Software as a Service (SaaS) platforms that eliminate the need for expensive custom coding. Second, we will introduce attendees to CIAS 3.0, highlighting key features and capabilities that allow the development of powerful screening, brief intervention, and referral applications. This will include review of embedded brief intervention templates—designed with input from top researchers using CIAS—that greatly facilitate learning and development in this platform. Third, we will consolidate information from the prior section by working with the group to collaboratively develop the beginnings of a short SBIRT application, which will remain on attendees’ dashboards for future reference or further development. (Note that all attendees will receive free access to CIAS 3.0 during the workshop so they can begin developing intervention content on their own dashboard during the workshop.) Fourth and finally, the presenters will share their extensive experience in dissemination and implementation CIAS-developed applications in healthcare settings. Workshop attendees will leave with the information and skills needed to start developing their own custom digital interventions using CIAS 3.0, including access to templates, training resources, and technical assistance as needed.

## NC-W02 Going beyond the referral to treatment: brief interventions as a tool for addressing harm reduction and whole-health care

### Heather A. Raley, Sadie M. Smith

#### Mosaic Group, Towson, MD, USA

##### **Correspondence:** Heather A. Raley (hraley@groupmosaic.com)

*Addiction Science & Clinical Practice* 2023, **19(Suppl 1):**NC-W02

**Description:** While SBIRT is preventative in nature, there is a responsibility to providers to take advantage of the opportunity for change, regardless of the patient’s readiness. Mosaic Group, a nationally-recognized consulting firm, has developed the Reverse the Cycle (RTC) model, adopted in over 70 hospitals, to help identify and respond to high-utilization patients who are using drugs and alcohol and leverages the SBIRT framework to provide harm reduction education and reduce a patient’s threat of harm through brief intervention. The model includes universal screening for risky substance use and peer response and recovery planning. Peer Recovery Coaches (PRCs), individuals with lived experience and in recovery from alcohol and/or substance use, are uniquely positioned to deliver brief interventions (BIs), leveraging this opportunity to create a space for future conversations and support, meeting patients where they are in their process and developing an appropriate plan to help the patient lead a healthier life. The BI serves as a means to identify not only substance-related needs, but also social needs that are creating barriers for patients to thrive.

True practice adoption begins with top organizational and clinical leadership buy-in, planning alongside a team of key hospital staff to champion the desired change, and fully embedding the model as part of the hospital workflow. This creates a team-based approach where PRCs are working alongside providers, nurses, and care management staff. This approach promotes timely harm reduction education, dissemination of tools for safer use, including community-based recovery coaching beyond discharge and the ability to collaborate with other members of the care team in real-time, shifting care to a whole-health model and seeing the patient as more than their substance use or chief complaint.

Mosaic Group has successfully implemented the Reverse the Cycle model in hospitals across 5 states and Washington, DC. The program provides a space to truly meet patients where they are, leading to a transformation of traditional "band-aid" care delivered in emergency departments. This shift results in longer-term relationships with patients and aims to reduce acute healthcare utilization while improving social risk factors such as housing and employment, psychiatric symptoms, and substance use. In this workshop, participants will collaborate to expand their view of a brief intervention beyond a referral to treatment, incorporating harm reduction and identification of the need for social resources that may lead to a healthier lifestyle.

Participants will work with partners and small groups using guided exercises to:

- Discuss how harm reduction principles and practices are applied in an emergency department setting. Practice “meeting patients where they are” using a brief intervention focused on harm reduction behavior.
- Utilize case studies to discuss how behavior change related to other presenting health issues in the emergency department can benefit from the brief intervention and practice integrating this “whole health” approach into a brief intervention.
- Identify how social determinants of health and other high-risk behaviors impact high risk substance use and develop open-ended questions that may elicit identification of social barriers and needs from the patient to be used in a brief intervention.

## NC-W03 Framing the conversation with youth: substance use in the wake of COVID-19

### Pam Pietruszewski, Emma Hayes

#### National Council for Mental Wellbeing, Washington, DC, USA

##### **Correspondence:** Pam Pietruszewski (pamp@thenationalcouncil.org)

*Addiction Science & Clinical Practice* 2023, **19(Suppl 1):**NC-W03

**Description:** The COVID-19 pandemic caused incredible disruption in the lives of young people during a period when they are already vulnerable in their growth, development, and identity. Between January 2021 and June 2022, the National Council for Mental Wellbeing (National Council) conducted a series of national assessments of youth ages 13–18 and youth-serving providers as part of the Getting Candid Initiative. These assessments examined the impact of COVID-19 on youth state of mind, knowledge of and access to substance use prevention programming, and effective messaging for communicating with youth about these areas. The assessment results revealed how this public health crisis continues to have lingering effects on the mental health and substance use of today’s youth. In fact, 75% of young people in the National Council’s 2021 assessment reported believing the pandemic will have a lasting impact on their generation’s mental health, and more than half reported believing it will have a lasting effect on their generation’s use of substances. Similar findings from the assessments became the basis for draft prevention messages that were tested and refined through focus groups and key informant discussions, then piloted in follow up assessments to youth. The National Council incorporated the finalized messages into a comprehensive message guide and toolkit to assist youth-serving providers engaging with youth in substance use prevention conversations, in light of potentially increased substance use in the wake of the pandemic. This workshop will be an interactive session designed to give participants the skills and tools to put this information into practice. The workshop will begin with a closer look at cumulative results from the two years’ worth of youth assessments and discussion groups. Presenters will demonstrate how these findings informed guidance for engaging youth in conversations around substance use and substance use prevention, including sharing information related to issues such as cannabis policy and the emergence of fentanyl. Presenters will then engage participants in a discussion of how to apply a communication pathway for building trust, gathering insight and framing the conversation using scenarios and youth collaborating videos. Additionally, the workshop will highlight toolkit resources such as the Findings Report, a Risks and Protective Factors Worksheet and On Demand Course.

## NC-W04 Brief interventions with people who inject drugs: harm reduction realities

### Melissa Floyd-Pickard, Michael Thull

#### UNC Greensboro, Greensboro, NC, USA

##### **Correspondence:** Melissa Floyd-Pickard (mftaylo2@uncg.edu)

*Addiction Science & Clinical Practice* 2023, **19(Suppl 1):**NC-W04

**Description:** This workshop will discuss the day-to-day operations of the Guilford County Solution to the Opioid Problem (GCSTOP), a harm reduction partnership between Guilford County Emergency Services and University of North Carolina Greensboro in operations since 2018. GCSTOP provides multi-faceted interventions, based in the principles of harm reduction to people dealing with opioid use disorders in Guilford County. Services include sterile syringe supplies to promote safer injection, NARCAN, fentanyl test strips, safer sex supplies and provision of HEP C/HIV testing and bridging to treatment as well as medication assisted treatment at no cost, and referral and transport to substance use detoxification and treatment if desired by the participant. Special care is given to justice-involved individuals who are incarcerated or have been recently released.

Attendees will gain knowledge of our brief interventions in and around Guilford County, often in mobile pop-up healthcare settings or in mobile visits to participants homes, sometimes meeting them in their motel rooms and tent encampments. They will learn about safer injection techniques, NARCAN administration, signs and symptoms of overdose, benefits of fentanyl and other drug testing, as well as the latest in medication interventions for opioid-use disorders (MOUD). Importantly, participants will learn how to build rapport with this vulnerable population and learn ways to promote partnership as well as barriers to authentic interaction to avoid. GCSTOP personnel will describe the beginnings of the program and especially the important aspects of partnering with local government, law enforcement and the academy. Multiple interventions along the social determinants of health will also be explored and discussed, including ways to balance transportation problems, soft-tissue and cardiac infections, food insecurity and unmet and trauma and mental health needs. The workshop leaders are skilled with multimedia presentations and present regularly on clinical issues through the exploration of case histories and best practices in the treatment of substance use disorders, in particular opioid use. The presentations will be interactive and questions will be taken throughout. Participants will also learn about diversity and equity issues in the harm reduction practice world and ways to increase participation by diverse individuals through the bridging of some common barriers.

## Oral presentations

## Best Abstract: NC-035 Are electronic brief interventions for unhealthy alcohol use based on norms and risk perception working as hypothesized?

### Joseph Studer^1,2^, John A. Cunningham^3,4^, Elodie Schmutz^1^, Jacques Gaume^1^, AngélineAdam^1^, Jean-Bernard Daeppen^1^, Nicolas Bertholet^1^

#### ^1^Addiction Medicine, Lausanne University Hospital and University of Lausanne, Lausanne, Switzerland; ^2^Service of Adult Psychiatry North-West, Department of Psychiatry, Lausanne University Hospital and University of Lausanne, Lausanne, Switzerland; ^3^National Addiction Centre, King’s College, London, United Kingdom; ^4^Center for Addiction and Mental Health, Toronto, ON, Canada

##### **Correspondence:** Nicolas Bertholet (nicolas.bertholet@chuv.ch)

*Addiction Science & Clinical Practice* 2023, **19(Suppl 1):**NC-035

**Background:** Many electronic interventions for unhealthy alcohol use are based on feedback on norms and risks for health. The hypothesis is that changing the perception of drinking norms and risks will result in changes in drinking. We investigated whether the effect of a smartphone intervention was mediated through these mechanisms.

**Material and methods:** 1770 students (Mean [SD] age = 22.35 [3.07]) with unhealthy alcohol use were randomized to receive access to a smartphone application or to a no-intervention control condition. The smartphone application provided normative feedback and personalized feedback on risks associated with drinking. A parallel mediation analysis was conducted to test whether the intervention effect was related to lower drinking (in standard drinks per week) at 6 months (adjusting for baseline values) through drinking norms and risk perceptions at 3 months (adjusting for baseline values).

**Results:** The total intervention effect was significant (b = − 0.87 [95% bootstrap confidence interval − 1.50; − 0.26]), indicating lower drinking at 6 months in the intervention group. The direct effect (i.e. controlling for mediators) was significant but smaller (b = − 0.75 [− 1.34; − 0.18]). The indirect effect was significant through drinking norms (b = − 0.12 [− 0.23; − 0.02]): The intervention was associated with lower drinking norms at 3 months (b = − 0.76 [− 1.33; − 0.16]), and norms at 3 months were associated with drinking at 6 months (b = 0.15 [0.08; 0.24]). The indirect effect through risk perception was not significant.

**Conclusion:** Drinking norms, but not risk perception, partially mediated the intervention effect on alcohol use, confirming one hypothesized mechanisms of action. These findings lend support for normative feedback interventions for unhealthy alcohol use.

## NC-001 Barriers to and facilitators of implementation of screening, brief intervention, and referral to treatment for risky substance use in pediatric primary care: a qualitative interview study

### Stacy Sterling

#### Center for Addiction and Mental Health Research, Division of Research, Kaiser Permanente Northern California, Oakland, California, USA

##### **Correspondence:** Stacy Sterling (stacy.a.sterling@kp.org)

*Addiction Science & Clinical Practice* 2023, **19(Suppl 1):**NC-001

**Background:** Detection of adolescent alcohol and other drug problems is critically important, and pediatric primary care is an opportune setting in which Screening, brief intervention and referral to treatment (SBIRT) can be delivered. In spite of endorsement by leading pediatric medical organizations, SBIRT has not been widely implemented in pediatric primary care, however, and there is relatively little research on factors contributing to its implementation.

**Material and methods:** Guided by the Consolidated Framework for Implementation Research (CFIR), we used qualitative data from Key Informant interviews (n = 20) with health system and community-based pediatric and specialty mental health and substance abuse treatment clinicians, and policymakers to examine determinants of SBIRT implementation in pediatric primary care. Analysis was conducted using NVivo software. Transcripts were independently coded, with coders blinded to the other’s codes during initial coding. Themes were identified based on the broad domains of the CFIR model: outer setting, inner setting, characteristics of the intervention, characteristics of the individuals involved, and process of implementation, with sub-categories reflecting greater nuance based on participant responses and informed by the extant literature. Coders discussed their understanding of themes, and differences were reconciled by consensus. Inter-rater reliability was calculated, and percentage coder agreement and a Kappa Coefficient calculated to measure inter-rater agreement by interview, node, and across the sample.

**Results:** Patient and family needs, comorbidity, confidentiality, growing awareness of the importance of behavioral health, stigma, marijuana legalization, provider time and competing priorities; the feasibility and accessibility of evidence-based screening instruments, adaptability of workflows, workforce training, linkages between relevant departments and organizations, leadership engagement, provider knowledge, skills and self-efficacy, and evidence quality were all identified as important determinants of SBIRT implementation.

**Conclusion:** Findings on implementation barriers and facilitators inform an implementation playbook focused on pragmatic steps to facilitate the implementation of adolescent SBIRT in health systems.

## NC-002 A decision theoretic model of optimal use of the AUDIT in SBI

### Arnie P. Aldridge, William Parish

#### RTI International, RTP, NC, USA

##### **Correspondence:** Arnie P Aldridge (aaldridge@rti.org)

*Addiction Science & Clinical Practice* 2023, **19(Suppl 1):**NC-002

**Background:** The optimal application of screening instruments for alcohol screening and brief intervention (SBI) depends not only on instrument misclassification for given score thresholds but on the SBI program’s setting and population. This leads to variability in both population outcomes and program economics that are important for SBI planning and policy.

**Material and methods:** We use simulation in a decision theoretic framework to assess the impact of misclassification on the net monetary benefits of SBI for alternative screen positive thresholds in the Alcohol Use Disorders Identification Test (AUDIT). The net monetary benefits of SBI which account for both the costs of SBI and the monetized benefits of SBI in terms of quality-adjusted life-years (QALYs). We focus on a hypothetical primary care SBI setting across a range of “true prevalence” assumptions for risky alcohol use. AUDIT performance characteristics and SBI effectiveness parameters are drawn from existing literature.

**Results:** As the value of QALYs increases the optimal AUDIT threshold score decreases from 5 to 4 at $3600/QALY in a full population. Optimal thresholds are different for men and women.

**Conclusion:** For reasonable ranges of SBI costs and QALY values, differences in the cost of misclassifying patients can lead to a different choice of AUDIT threshold screen positive scores.

## NC-004 Brief intervention for alcohol misuse among people living with HIV: a systematic review and meta-analysis

### Abhishek Ghosh^1^, Geetesh K. Singh^2^, Nidhi Yadav^1^, Pranshu Singh^3^, Sanjana Kathiravan^1^

#### ^1^Drug Deaddiction and Treatment Centre, Department of Psychiatry, Postgraduate Institute of Medical Education and Research, Chandigarh, India; ^2^SCBS | Rashtriya Raksha University (An Institute of National Importance) Lavad, Gandhinagar, Gujarat, India; ^3^Department of Psychiatry, All India Institute of Medical Sciences, Jodhpur, Rajasthan, India

##### **Correspondence:** Abhishek Ghosh (ghoshabhishek12@gmail.com)

*Addiction Science & Clinical Practice* 2023, **19(Suppl 1):**NC-004

**Background:** Nearly one-third people living with HIV (PLHIV) have alcohol misuse. In addition to the harm directly caused by it, alcohol misuse negatively affects the course and outcome of HIV. We wanted to determine the effect of brief intervention (BI) on alcohol and HIV outcomes.

**Material and methods:** We included clinical trials on BI in adults with harmful or hazardous alcohol use, published between 1990 and September 2022; however, only randomized clinical trials (RCTs) were included in the meta-analysis. We searched MEDLINE, Cochrane Central Register of Controlled Trials, Clinical Trials.Gov, and the World Health Organization’s International Clinical Trials Registry Platform databases. We used Relative Risk (RR) or Odds Ratio (OR) and standardized mean difference (SMD) for dichotomous and continuous outcomes. We evaluated study quality by Cochrane’s risk of bias assessment tool.

**Results:** Eighteen studies (N = 4737, Female = 30.3%) were included in the narrative synthesis; meta-analysis was performed on 13 studies. BI significantly reduced the drinks per drinking day (N = 5, Hedge’s g = − 0.45, 95% CI = − 0.58, − 0.32) and the number of heavy drinking days (N = 4, Hedge’s g = − 0.81, 95% CI = − 0.94, − 0.67) at six-months. However, BI did not significantly change the alcohol risk scores and risk of transition to harmful/hazardous use. BI did not improve the adherence to Anti-Retroviral Therapy or increase rates of viral suppression. The proportion of people retained in study was not different between BI and controls (N = 10, RR = 1.01, 95% CI = 0.98, 1.04). Most importantly, BI reduced the odds of mortality by 42 percent (N = 7, OR = 0.58, 95% CI = 0.34, 0.99, low-risk for publication bias) at 6–12 months. Out of the 15 RCTs, six showed a low risk of bias, and two showed a high risk; others have some concerns.

**Conclusion:** BI is a promising tool for alcohol misuse among PLHIV. It must be integrated into HIV services and scaled up.

## NC-013 Results and perspectives of the implementation of training of trainers on screening and brief intervention and referral to treatment (SBIRT) for tobacco use in France

### Marianne Hochet, Nicolas Bonnet

#### ^1^RESPADD, Villejuif, Ile-de-France, France

##### **Correspondence:** Marianne Hochet (marianne.hochet@respadd.org)

*Addiction Science & Clinical Practice* 2023, **19(Suppl 1):**NC-013

**Background:** The prevalence of smoking is still very high in France despite all the public health actions led during past years: 25% of people aged between 18 and 75 years old were daily smokers in 2022. One action taken in France is to encourage and support hospitals and health services in becoming tobacco-free. In order for this strategy to be efficient, healthcare professionals must be trained to screen smokers and support them quitting. Thus, the French addiction prevention network, RESPADD, which is the national coordinator of this strategy, has implemented training of trainers to quickly disseminate knowledge and know-how.

**Material and methods:** Between 2018 and 2022, 13 training sessions have been set up, one in each French region, and 252 participants were trained to become themselves trainers to then disseminate SBIRT for tobacco use for healthcare professionals. To have feedback on these training sessions and to know if the new trainers did implement their own training sessions, we send questionnaires by emails to the 252 new trainers at the end of 2022. The questionnaire is an evaluation of the impact of these training sessions: on nicotine substitutes prescription, on SBIRT systematic use and on training session implementation.

**Results:** We received 70 answers to this questionnaire. The results showed more than 75% of participants are now using SBIRT in their daily work, more than 41% are prescribing nicotine substitutes and more than 35% are now implementing their own training sessions for their colleagues or for other health structures. 234 training sessions were then set leading to a total of around 2300 healthcare professionals trained.

**Conclusion:** To train trainers is very useful to disseminate rapidly and widely SBIRT and to increase the community of healthcare professionals involved in this care and support method.

## NC-015 Screening and brief intervention rates before and after COVID-19 onset in an US integrated healthcare system with systematic alcohol SBIRT in adult primary care

### Felicia W. Chi^1^, Vanessa A. Palzes^1^, Yun Lu^1^, Thekla B. Ross^1^, Constance Weisner^1^, Derek D. Satre^1,2^, Stacy A. Sterling^1^

#### ^1^Division of Research, Kaiser Permanente Northern California, Oakland, California, USA; ^2^Department of Psychiatry and Behavioral Sciences, Weill Institute for Neurosciences, University of California, San Francisco, California, USA

##### **Correspondence:** Felicia W. Chi (felicia.w.chi@kp.org)

*Addiction Science & Clinical Practice* 2023, **19(Suppl 1):**NC-015

**Background:** Unhealthy alcohol use is a public health problem with significant health, social and economic impacts. Screening, Brief Intervention, and Referral to Treatment (SBIRT) in adult primary care is an evidence-based approach enabling early identification and intervention of unhealthy alcohol use. During the COVID-19 pandemic, alcohol consumption and unhealthy use has increased for specific subgroups of the population in certain countries, yet little is known about the impact of the pandemic on alcohol screening and brief intervention. Using electronic health record data in an US integrated healthcare system that implemented systematic alcohol SBIRT in adult primary care in 2013, this observational study evaluated the change in monthly alcohol screening and brief intervention rates in the context of the COVID-19 pandemic, from January 2019 through.

**Material and methods:** The study setting was Kaiser Permanente Northern California. We examined level and slope change post COVID-19 onset in screening rates among patients with primary care visits, and brief intervention rates among those screened positive for unhealthy alcohol use, by conducting interrupted time series analysis using population-level aggregated data.

**Results:** Alcohol screening rates dropped substantially immediately after COVID-19 onset but steadily increased. Brief intervention rates among those screened positive also dropped immediately after COVID-19 onset and increased after that; however, they did not bounce back to pre-COVID-19 level as of end of 2022.

**Conclusion:** The decline in both screening and brief intervention was likely due to competing pandemic-related priorities and the transition to telemedicine. Innovative workflows, strategies and support (e.g., pre-visit screening via patient portals and e-delivery of BI) are needed to avoid future interruptions of SBIRT for unhealthy alcohol use in the post-COVID-19 era. Moreover, our findings underscore the need for “booster” trainings of clinicians to support ongoing sustainment of systematic SBIRT programs both generally and in response to system disruptions.

## NC-018 Brief intervention: qualitative study of the extent of knowledge among health workers in primary care centers in Nigeria

### Fatima Abiola Popoola, Sanusi Muhammad Salisu

#### National Drug Law Enforcement Agency, FCT Abuja, Nigeria

##### **Correspondence:** Fatima Abiola Popoola (abiolaodus9@gmail.com)

*Addiction Science & Clinical Practice* 2023, **19(Suppl 1):**NC-018

**Background:** The implementation of Brief Intervention in primary health treatment centers in Nigeria is an uncommon phenomenon; considering the inadequacies of treatment resources, the primary health care centers located within the communities are the first point of call for persons needing healthcare services especially in the cases of emergency. There have been some cases death of PWUD but it has not been established if these were due to substance use complications or lack of immediate interventions, with adequate information on available interventions, where and how to access treatment, the importance of these groups to assist in substance use prevention and treatment would be achieved.

**Material and methods:** This is a qualitative research using the interview method to elicit information from 120 primary health workers comprising of 60 Nurses, 30 resident doctors and 35 community pharmacists working in primary health care in some selected centers in Nigeria. The interviews were carried out on one-on-one basis and it focused on the extent of knowledge on BI, the responses were collated as results for this study.

**Results:** The findings revealed that of the 120 health workers interviewed 30% have knowledge of BI, 23% have acquired training on BI in the past but have never used it, 31% have never heard of BI and 24% are willing to be trained if given the opportunity. Findings also clarified the need for patients to feel free to discuss their drug problems with health workers when they visit the community health care centers.

**Conclusion:** Health workers in the community to offer support to PWUD, treatment should go beyond regular health issues, interventions for drug related problems should be taken seriously to improve quality of care within the community.

## NC-024 Does screening mode matter? Computer self-administered versus clinician-administered screening of youth substance use in a large pediatric primary care database

### Madison M. O’Connell^1^, Barbara Howard^2,3^, Raymond Sturner^2,4^, Julia Plumb^1^, Cynthia Tran^3^, Lydia A. Shrier^1,5^, Sion Kim Harris^1,5^

#### ^1^Division of Adolescent/Young Adult Medicine, Boston Children’s Hospital, Boston, MA, USA; ^2^Pediatrics, The Johns Hopkins University School of Medicine, Baltimore, USA; ^3^CHADIS, Inc., 6017 Altamont Place, Baltimore, MD, USA; ^4^Center for Promotion of Child Development Through Primary Care, Baltimore, MD, USA; ^5^Department of Pediatrics, Harvard Medical School, Boston, MA, USA

##### **Correspondence:** Madison M. O'Connell (madison.oconnell@childrens.harvard.edu)

*Addiction Science & Clinical Practice* 2023, **19(Suppl 1):**NC-024

**Background:** Early detection and intervention through universal youth screening can help decrease risk of problematic substance use; however, screening mode (computer self-administered [SA] vs. clinician-administered [CA]) may influence detection and, therefore, clinical response.

**Material and methods:** We analyzed SU screening responses collected between 2018 and 2022 from 12- to 20-year-olds seen at 314 U.S. pediatric practices utilizing the CHADIS online clinical process support system. The CRAFFT screen assessed past-12-month alcohol, cannabis, and other SU (“anything else to get high”). We compared SU rates by screening mode (SA vs. CA), using logistic regression modeling with GEE (to account for data clustering within practices and patients) to compute adjusted odds ratios and 95% confidence intervals (AOR, 95% CI). Models controlled for age (days), sex, U.S. region, and data year. We stratified analyses by age group (12–13; 14–15; 16–17; 18–20 years). We used the Benjamini-Hochberg procedure to correct for multiple comparisons.

**Results:** Youth (N = 130,688) were 51.5% females; 24.6% age 12–13, 29.5% 14–15, 28.7% 16–17, 17.2% 18–20 years; 49.1% from South, 34.7% Northeast, 6.9% Midwest, 9.4% West. Screening mode was 74.9% SA and 25.1% CA. Compared to CA, SA screening was associated with significantly higher adjusted odds of reporting any SU (AOR, 95% CI by age group: 12–13 1.75, 1.43–2.15; 14–15 1.21, 1.11–1.33; 16–17 1.32, 1.24–1.41; 18–20 1.49, 1.39–1.58). In general, alcohol and cannabis demonstrated similar patterns of significance. Report of other SU did not differ by screening mode in any age group, but prevalence was low (0.1–2.1%). Most differences remained significant after correction for multiple comparisons.

**Conclusion:** Self-administered screening was associated with higher prevalence of SU than clinician-administered screening. Youth SU disclosure may be lower in clinician-administered interviews due to factors such as social desirability or confidentiality. Pediatric primary care practices may wish to consider self-administered SU screening to facilitate youth SU detection and guide clinical response.

## NC-025 Developing context-specific intervention components for alcohol reduction within the Drink Less app

### Melissa Oldham^1^, Abigail K. Stevely^2^, Claire Garnett^3^, John Holmes^2^, Andrew Jones^4^, Larisa Dinu^1^

#### ^1^Department of Behavioural Science and Health, University College London, UK; ^2^School of Health and Related Research (ScHARR), University of Sheffield, UK; ^3^School of Psychological Science, University of Bristol, Bristol; ^4^School of Psychology, Liverpool John Moores University, Liverpool, UK

##### **Correspondence:** Melissa Oldham (m.oldham@ucl.ac.uk)

*Addiction Science & Clinical Practice* 2023, **19(Suppl 1):**NC-025

**Background:** The contexts in which people drink (e.g. location, company) and reasons for drinking (e.g. to celebrate, to relax) are highly variable. Personalising intervention strategies to individuals’ drinking contexts may be more effective than a ‘one-size-fits-all’ approach. This project aims to improve the efficacy of the established ‘Drink Less’ app by (a) developing a drinking diary which measures drinking contexts and (b) developing and refining context-specific ‘self-monitoring and feedback’ and ‘action planning’ intervention components.

**Methods:** To establish how to collect data on drinking contexts, an experimental mixed-methods study was conducted. Participants were recruited using Prolific and randomly assigned to use one of two adapted versions of the Drink Less app for 14 days. Version 1 (n = 31) included location, motivation and company tags that participants added to drink records in the drinking diary. Version 2 (n = 31) included an occasion type list for participants to select from when adding drink records. Primary outcome variables were data quality and usability. Interviews with a subsample of 16 participants focused on acceptability. Two context-specific intervention components were then co-developed with reference to the Capability-Opportunity-Motivation-B (COM-B) model of behaviour change and refined via expert synthesis, PPI consultation and six focus groups with potential app users.

**Results:** Both versions of the context specific drinking diary were rated similarly in terms of usability and acceptability. Data quality was slightly higher for version 2 with the occasion types. Context-specific intervention messaging was then iteratively developed and was evaluated and ranked by wider experts in intervention development and behaviour change and PPI groups. The highest ranked messaging options will be evaluated in six focus groups resulting in two context-specific intervention components, ‘self-monitoring and feedback’ and ‘action planning’.

**Conclusion:** This study will refine an existing digital intervention to provide tailored advice based on the contexts in which individuals drink.

## NC-027 Addressing alcohol, marijuana, and substance use in a U.S. multi-state reproductive healthcare organization: does patient use differ in states with legalized access to marijuana?

### Faith O. Green^1^, Corrie Whitmore^2^, Megan Waddell^1^, Diane K. King^1^

#### ^1^University of Alaska, Anchorage Centers for Behavioral Health Research and Services, Anchorage, Alaska, USA; ^2^University of Alaska, Anchorage College of Health, Anchorage, Alaska, USA

##### **Correspondence:** Faith Ozer Green (fozer@alaska.edu)

*Addiction Science & Clinical Practice* 2023, **19(Suppl 1):**NC-027

**Background:** With changes in legal access to marijuana in the U.S., self-reported drug use patterns have shifted, fueling concerns that marijuana use during pregnancy may adversely affect prenatal development. With this in mind, we explored variations in marijuana, alcohol and other drug use among patients receiving preventive services from one of 33 health centers located in Alaska, Idaho, Indiana, Kentucky, and Washington. The participating healthcare organization is part of a national implementation project to incorporate universal Screening and Brief Intervention into clinical operations during preventative wellness visits, to prevent alcohol- and substance-exposed pregnancies.

**Material and methods:** Data were extracted from electronic health records for adults assigned-female-at-birth, aged 18–44, who completed wellness visits between October 1, 2022 and February 28, 2023.

**Results:** Seventy-four percent of the sample (N = 3052) received services in states with legalized recreational marijuana. Differences in reported drug use (positive DAST), marijuana use (single item), and “risky” alcohol use (US AUDIT > 6; i.e., above “safe” consumption levels) were statistically significant (p < 0.01); with a higher percentage reporting use in states that legalized recreational marijuana. Marijuana use in ‘legal’ states was especially high (28.6% versus 17.4%), and higher percentages of use held for other drugs (19.2% versus 6.8%) and for “risky” alcohol use (9.3% versus 5.7%). There were no significant differences in overall alcohol use between these two groups.

**Conclusion:** Patients seeking healthcare in states with legalized recreational marijuana self-reported higher rates of risky alcohol, drug, and marijuana use, compared to states where marijuana is illegal, a finding consistent with other recent studies. State policies, which influence drug use and patient willingness to self-report, have implications for identifying, communicating and addressing the health risks associated with these diverse substances. Future research should explore the potential impact of legalization on patients’ disclosure of use and provider communication around health risks.

## NC-028 Baseline results from EvidenceNOW: managing unhealthy alcohol use in primary care

### Brett Harris, Tracy McPherson, Nora Marino, Adrienne Call, Alexandria Figueroa, Jenna Sirkin

#### NORC, University of Chicago, Chicago, IL, USA

##### **Correspondence:** Tracy McPherson (mcpherson-tracy@norc.org)

*Addiction Science & Clinical Practice* 2023, **19(Suppl 1):**NC-028

**Background:** Excessive alcohol use is a leading cause of preventable death, responsible for 1 in 5 deaths among U.S. adults. To address excessive alcohol use, the Agency for Healthcare Research and Quality launched EvidenceNOW: Managing Unhealthy Alcohol Use in Primary Care in 2019. This initiative funds six grantees to disseminate evidence-based approaches for managing unhealthy alcohol use (UAU) in primary care including screening, brief intervention, and referral to treatment (SBI/RT) and medication for addiction treatment (MAT). An analysis of baseline data from practices across the country was conducted to assess needs and motivations for engaging in the initiative and implementing SBI/RT and MAT.

**Material and methods:** Over 200 practices submitted baseline quantitative data on practice characteristics, policies and procedures, and delivery of SBI/RT and MAT between February and November 2022. Forty-two key informant interviews were conducted with grantees and 12 with practices between June 2021 and October 2022 to gather qualitative data on the experiences of grantees and practices.

**Results:** Most practices (97%) had not fully implemented all components of SBI/RT at baseline. Across all practices, only 38% reported screening more than a quarter of patients, and 76% did not provide brief intervention to any patients at all. Most did not provide MAT. Practices reported that receiving practice facilitation was a key motivator for participating in the initiative, offering them the opportunity to learn new skills, standardize their approach to addressing UAU with evidence-based care, and engage in quality improvement.

**Conclusion:** Baseline findings indicate that, although many practices were attempting to address UAU in some way, a comprehensive, systematic approach was uncommon prior to participating in the initiative. Practices were motivated by the opportunity to standardize and improve their approach to addressing UAU, suggesting the importance of this motivator in increasing uptake of SBI/RT and MAT.

## NC-029 Clinician perspectives on the feasibility of implementing screening and brief interventions in secondary care cardiology services in Sweden: a qualitative study

### Paul Welfordsson^1^, Anna-Karin Danielsson^1^, Caroline Björck^2,3^, Bartosz Grzymala-Lubanski^3,4^, Sara Wallhed Finn^1,5,6^

#### ^1^Department of Global Public Health, Karolinska Institutet, Stockholm, Sweden; ^2^Department of Women’s and Children’s Health, Akademiska sjukhuset, Uppsala University, Uppsala, Sweden; ^3^Centre for Research and Development, Region Gävleborg, Gävle, Sweden; ^4^Department of Public Health and Clinical Medicine, Umeå University, Umeå, Sweden; ^5^Unit of Clinical Alcohol Research, Institute of Clinical Research, University of Southern Denmark, Odense, Denmark; ^6^Centre for Dependency Disorders, Stockholm, Sweden

##### **Correspondence:** Paul Welfordsson (paul.welfordsson@ki.se)

*Addiction Science & Clinical Practice* 2023, **19(Suppl 1):**NC-029

**Background:** Cardiovascular disease (CVD) is the leading contributor to disability-adjusted life years and mortality. Secondary prevention strategies in cardiology often address behavioral risk factors such as physical inactivity and tobacco use, yet there has been comparatively little focus on alcohol prevention. The aim of this study was to explore clinician perspectives on the feasibility of implementing screening and brief interventions (SBI) in secondary care cardiology services in Sweden.

**Material and methods:** A qualitative study was conducted at district general hospitals in three geographically diverse regions of Sweden. Individual interviews were completed with a hierarchical, purposive sample of 24 clinical cardiology staff (9 doctors, 11 nurses and 4 assistant nurses). A variety of experience and care levels (outpatient clinic, cardiology ward, intensive care) were represented. Sample size was based upon an assessment of information power. Open-ended, semi-structured interview questions focused broadly on clinicians’ views regarding the feasibility of implementing SBI in cardiology settings, including perceived implementation barriers and facilitators. Reflexive thematic analysis was used, with deductive coding based upon the Theoretical Domains Framework and COM-B system for understanding health practitioner behaviours. Ethical approval was obtained (2021-06819-01).

**Results:** Clinicians’ views ranged from cautious optimism to overt pessimism. Perceived implementation barriers included a lack of knowledge about SBI and doubts about self-reported measures of alcohol use (capability), concerns about the environmental context and resource limitations within acute cardiology settings (opportunity), and perceptions that SBI was outside of cardiology clinicians’ professional role (motivation). However, clinicians perceived hazardous alcohol use to be an important health issue. Effective communication between clinicians and patients was seen as a facilitating factor.

**Conclusion:** Clinicians’ views on the feasibility of implementing alcohol interventions in cardiology vary. Overall, SBI was perceived as important, but several barriers were identified. These may threaten the success and sustainability of SBI in cardiology settings.

## NC-030 Can a national mass media campaign increase treatment-seeking for alcohol use disorders?

### Sara Wallhed Finn^1^, Anna Mejldal^2^, Anette Søgaard Nielsen^3^

#### ^1^Department of Global Public Health, Karolinska Institutet, Stockholm, Sweden; ^2^Unit of Clinical Alcohol Research, Institute of Clinical Research, University of Southern Denmark, Odense, Denmark; ^3^Psychiatric Hospital, University Function, Region of Southern, Odense, Denmark

##### **Correspondence:** Sara Wallhed Finn (sara.wallhed-finn@ki.se)

*Addiction Science & Clinical Practice* 2023, **19(Suppl 1):**NC-030

**Background:** A minority of individuals with alcohol use disorders (AUD) seek treatment. Stigma is one important barrier. In Denmark, a nationwide mass media campaign, “RESPEKT”, aiming to increase treatment seeking, has been broadcasted since year 2015. Little is known about effects of this type of intervention. The aim is to summarize two interlinked studies: (1) effects of video materials used in the campaign on stigma and motivation to change alcohol use and motivation to seek treatment; and (2) associations between campaign periods and treatment seeking for AUD.

**Materials and methods:** Study one: Three-armed double blind randomized controlled study. Participants were Danish adults (n = 655), randomized 1:1:1 ratio, to view a video. Video 1 and 2 were part of the “RESPEKT” campaign and video 3 was a control condition. Outcomes were analyzed with mixed effects linear regression.

Study two: Interrupted time-series analysis, using national registers of the total Danish population, year 2013 to 2018. Exposure: Campaign periods year 2015 to 2018. Outcome: Treatment seeking defined as specialized AUD treatment or filled prescription of AUD pharmacotherapy. Analysis: Segmented negative binomial regression.

**Results:** (1) Video 1 and 3 decreased public stigma, while video 2 increased stigma. All videos reduced motivation to change alcohol use. No effects were seen on motivation to seek treatment. (2) The results show no association between the campaign periods and treatment seeking.

**Conclusions:** Videos can have immediate effect on level of public stigma. Neither the videos nor campaign periods were associated with change in motivation to seek treatment or treatment seeking. To avoid adverse effects in future interventions, use of theoretical frameworks and stakeholder involvement is emphasized. We suggest focusing on earlier steps of the treatment seeking process, as problem recognition, to increase treatment seeking. There is a great need to develop other ways to narrow the treatment gap for AUD.

## NC-031 Substance use research education and training program (SARET)

### Mia Malone^1^, Kathleen Hanley^1^, Jennifer McNeely^1^, Danielle Ompad^2^, Lance Keene^3^, Selena A Gilles^4^, Lukasz Witek^5^, Marc N. Gourevitch^1^

#### ^1^New York University Grossman School of Medicine, New York, NY, USA; ^2^New York University School of Global Public Health, New York, NY, USA; ^3^New York University School of Social Work, New York, NY, USA; ^4^New York University Rory Meyers College of Nursing, New York, NY, USA; ^5^New York University College of Dentistry, New York, NY, USA

##### **Correspondence:** Mia Malone (mia.malone@nyulangone.org)

*Addiction Science & Clinical Practice* 2023, **19(Suppl 1):**NC-031

**Background:** The SARET program annually recruits approximately 15 graduate-level students from NYU’s Schools of Medicine, Nursing, Social Work, Dentistry, and Global Public Health. Its Visiting Mentor Development Program (VMDP) component enrolls a similarly interprofessional group of approximately 5 visiting faculty mentors each summer.

**Methods:** We regularly evaluate the long-term impact of student participation in SARET utilizing a survey, administered every five years, and conduct annual bibliometric analyses of all program participants. The survey assesses participants’ subsequent involvement in substance use research, career trajectories, and SARET’s influence on their substance use knowledge. Bibliometric analysis tracks participants’ presentations, publications, and extramural funding. For VMDP participants, quarterly phone calls assess their progress in developing and implementing mentored research programs in their home institutions. Additionally, we track completions of SARET’s online curriculum modules, to examine uptake, interprofessional interest, and educational efficacy.

**Results:** Students: Since inception in 2007, 166 students participated in SARET. Participants have published substantially in SUD research, including 99 peer-reviewed articles and 49 oral/poster presentations at national meetings. Visiting mentors: VMDP has hosted 20 visiting mentors, of which three (15%) have already initiated new inter-disciplinary SU mentored research programs. Two VMDP participants developed a substance use research course in their undergraduate program and are working on creating a SU research minor. Modules: Of 36,000 total module completions, 23,000 have been at NYU and over 13,000 at other institutions.

**Conclusion:** The SARET program has stimulated enduring substance use research among health and public health trainees, and resulted in a number of early successes among participants. VMDP shows promise as a model for disseminating the development of interdisciplinary substance use mentored research programs at other institutions. SARET’s free online modular curriculum increases access to substantive research and treatment educational materials that can be utilized to foster interest in substance use research at academic institutions.

## NC-032 Providing culturally safe interventions to prevent alcohol-exposed pregnancies

### Megan Waddell^1^, Faith O. Green^1^, Corrie Whitmore^2^, Janice Vendetti^3^, Bonnie McRee^3^, Karen L. Steinberg^3^, Diane K. King^1^

#### ^1^University of Alaska, Anchorage Centers for Behavioral Health Research and Services, Anchorage, Alaska, USA; ^2^University of Alaska, Anchorage College of Health, Anchorage, Alaska, USA; ^3^University of Connecticut Medical School, Storrs Connecticut, USA

##### **Correspondence:** Faith Ozer Green (fozer@alaska.edu)

*Addiction Science & Clinical Practice* 2023, **19(Suppl 1):**NC-032

**Background:** Healthcare providers are well-positioned to discuss risks and options for preventing alcohol-exposed pregnancy (AEP). Patient-centered approaches to providing information may overlook systemic and historical biases, cultural beliefs about pregnancy, and patient concerns about contraception safety, diminishing trust and receptivity to recommendations, and exacerbating health disparities.

**Material and methods:** We explored consistency of delivering universal alcohol screening and brief intervention (SBI) services to patients ages 18–49 accessing care at two Planned Parenthood affiliates: Planned Parenthood of Southern New England (PPNSE) and Planned Parenthood of the Great Northwest, Hawaii, Alaska, Indiana, Kentucky (PPGNHAIK). Race, ethnicity, contraception effectiveness (i.e., ≤ 88% effective), and alcohol SBI data were extracted from electronic health records for patient wellness visits completed between 6/1/20–10/31/22. Data were analyzed to identify differences in risk for an AEP and receipt of SBI services across race/ethnicity groups.

**Results:** Within PPSNE (*N* = 10,303), Black patients were at highest risk for an AEP with a greater percentage reporting alcohol use combined with less effective contraception (*p* < 0.001). Whites had the highest screening completion rates (*p* < 0.001); brief intervention rates did not differ by race/ethnicity. Within PPGNHAIK (*N* = 17,695), all groups reported similarly low levels of alcohol use; however, Black and Multi-racial patients were at higher risk for an AEP due to a greater percentage using less effective contraception (*p* < 0.01). Screening rates did not vary; however, Asian/Pacific Islander patients were less likely to receive a brief intervention than other groups (*p* < 0.01).

**Conclusion:** Despite universal delivery of SBI, intervention effectiveness requires identifying whether patients with higher risk factors are being offered information and advice in an environment that fosters trust and respects their choices for contraception and alcohol use. Cultural safety can be improved by addressing individual, professional and systemic biases and power differentials that undermine the effectiveness of reproductive health counseling.

## NC-033 Brief interventions for reducing alcohol consumption in workers of city hall

### Tereza Barroso, Lícia Teixeira, Daniela Pinto

#### Health Sciences Research Unit: Nursing, Nursing School of Coimbra, Coimbra, Portugal

##### **Correspondence:** Tereza Barroso (tbarroso@esenfc.pt)

*Addiction Science & Clinical Practice* 2023, **19(Suppl 1):**NC-033

The primary objective of brief interventions (BIs) is to detect hazardous and harmful alcohol consumption and encourage behavior change.

**Objectives:** To assess the effect of BIs in reducing alcohol consumption in workers of city hall.

**Methodology:** A pre-experimental study, with assessment before and after the intervention (5 months), was conducted with 55 users (single group) (22 famules and 33 males; average age 48,9. The Alcohol Use Disorder Identification Test (AUDIT) was used. The BIs were developed by mental health nurse.

**Results:** At baseline, 87,3% of users were at risk level I and 12,7% were at risk level II. The follow-up (5 months after BIs), will be conducted at may month.

**Conclusion:** We expect that BIs reduced and stabilized the risk levels of alcohol consumption, reinforcing the importance of their application in primary care.

## NC-034 Assessing the landscape of adolescent screening, brief intervention, and referral to treatment (SBIRT) in Colorado: implications for workforce development and improving practice

### Giana Calabrese^1^, Tracy McPherson^1^, Abby Mariani^1^, Nora Marino^1^, Carolyn Swenson^2^, Adam Musielewicz^2^, Elizabeth Pace^2^, Ellen Velez^2^

#### ^1^NORC at the University of Chicago, Bethesda, MD, USA; ^2^Peer Assistance Services, Inc., Denver, CO, USA

##### **Correspondence:** Tracy McPherson (mcpherson-tracy@norc.org)

*Addiction Science & Clinical Practice* 2023, **19(Suppl 1):**NC-034

**Background:** Youth-serving health professionals can effectively identify, reduce, and prevent alcohol and other substance use through the evidence-based model, Screening, Brief Intervention, and Referral to Treatment (SBIRT). This study examines the implementation of adolescent SBIRT among Colorado health professionals in a variety of disciplines and roles, and identifies training and technical assistance opportunities to strengthen the workforce and optimize SBIRT in practice.

**Material and methods:** Practitioners serving youth ages 11–25 (N = 205) in primary/integrated care, hospitals, and school-based settings completed an online survey. Quantitative and qualitative data collection occurred October 2022–April 2023. Snowball sampling was used to recruit practitioners through professional associations, educational and public health agencies, and health systems. Key informant interviews were conducted with medical and behavioral health professionals.

**Results:** Only 11% of practitioners reported implementing all components of SBIRT (i.e., screening, brief intervention/motivational interviewing, hand off/arrange referral, schedule/provide follow-up). Among the 74% who reported screening, 60% did not use a validated tool. Among those who reported conducting brief intervention, over 55% did not utilize common, core elements of widely accepted models of brief intervention such as providing feedback, using motivational interviewing strategies, and setting behavior change goals. On average, practitioners reported low to moderate knowledge and confidence delivering SBIRT with youth, with nurses and professionals in school settings feeling the least knowledgeable and confident. Almost all practitioners reported the need for technical assistance, training, and/or resources to improve SBIRT practice.

**Conclusion:** Given the high rates of substance use among youth in Colorado, it’s imperative to strengthen the knowledge, confidence, and skills of the workforce, as well as the implementation of SBIRT services. Findings shed light on opportunities for healthcare, public health, and school settings to mobilize existing SBIRT training and technical assistance resources to optimize youth substance use prevention across Colorado.

## NC-037 Comparing under-reporting and correctly-reporting of risky drug use among primary care patients in federally qualified health centers

### Cristina Batarse^1^, Daisy Hernandez-Casas^1^, Quynh T. N. Vo^1^, Maria Vasquez^1^, Amanda Sirisoma^1^, Melvin W. Rico^1,4^, Stephanie Sumstine^2^, Leticia Cazares^1^, Dallas Swendeman^2^, Lillian Gelberg^1,3^

#### ^1^Department of Family Medicine, David Geffen School of Medicine at UCLA, Los Angeles, California, USA; ^2^Department of Psychiatry and Biobehavioral Sciences, David Geffen School of Medicine at UCLA, Los Angeles, California, USA; ^3^Department of Health Policy & Management, Fielding School of Public Health, UCLA, Los Angeles, California, USA; ^4^Charles R. Drew University of Medicine and Science, Los Angeles, California, USA

##### **Correspondence:** Cristina Batarse (cristinabatarse@gmail.com)

*Addiction Science & Clinical Practice* 2023, **19(Suppl 1):**NC-037

**Background:** COVID-19 accelerated remote patient substance use screening via telehealth, mobile-web, and mail-based biomarkers. We assessed concordance of substance use between mail-based self-collected UDS and self-reports among adult FQHC primary care patients in LA County.

**Methods:** Customized urinalysis with blinded purpose and results indicators were mailed to patients screening positive for moderate risk drug use on the self-administered WHO ASSIST (score 4–26) via mobile-web for a SUD prevention intervention trial. Patients were instructed that urine results wouldn’t be included in medical records and only used to compare to questionnaire responses. Patients texted photos of urinalysis to research staff using mobile phones. We assess UDS completion rates, drug use under-reporting, and associated factors.

**Results**: Of 98 eligible patients, 78 (80%) provided UDS results with 94% being readable. Sixty-one tested positive for at-least one drug. Comparing self-reports to urinalysis, the correct self-reporting vs underreporting were: cannabis 48 (62%) vs. 1 (1%), tobacco 18 (23%) vs. 5 (6%), methamphetamine 5 (6%) vs. 1 (1%), amphetamine 1 (1%) vs. 10 (13%), and morphine 2 (3%) vs. 4 (5%). Overall, 22 (28%) under-reported any drug use. In KIND analyses, under-reporting was associated with female gender, incarceration history, and homelessness (odd ratios 2.2, 1.9, 2.5 respectively; P < 0.05).

**Conclusions:** Mail-based self-collected UDS with results shared digitally is feasible among a majority of FQHC patients self-reporting moderate risk drug use. Under-reporting was low and varied by substance. Patients with prior life experiences involving drug use screening may be more reluctant to participate and need additional support.

## NC-038 Developing a protocol for substance use screening and referral to treatment for FQHC primary care patients with high-risk substance use

### Quynh T. N. Vo^1^, Cristina Batarse^1^, Daisy Hernandez-Casas^1^, Melvin W. Rico^1,4^, Stephanie Sumstine^2^, Leticia Cazares^1^, Jack Tsai^5^, Armen Arshakyan^6^, Dallas Swendeman^2^, Lillian Gelberg^1,3^

#### ^1^Department of Family Medicine, David Geffen School of Medicine at UCLA, Los Angeles, California, USA; ^2^Department of Psychiatry and Biobehavioral Sciences, David Geffen School of Medicine at UCLA, Los Angeles, California, USA; ^3^Department of Health Policy & Management, Fielding School of Public Health, UCLA, Los Angeles, California, USA; ^4^Charles R. Drew University of Medicine and Science, Los Angeles, California, USA; ^5^The Children’s Clinic, “Serving Children and Their Families” Long Beach, California, USA; ^6^Saban Community Clinic, Los Angeles, California, United States

##### **Correspondence:** Cristina Batarse (cristinabatarse@gmail.com)

*Addiction Science & Clinical Practice* 2023, **19(Suppl 1):**NC-038

**Background:** Federally Qualified Health Centers (FQHCs) provide opportunities to screen patients and offer referrals for substance use disorder (SUD) treatment. Little is known about the referral to treatment (RT) process, and no standardized protocol has been accepted across FQHCs. The QUIT-Mobile study is a NIDA-funded RCT that screens primary care patients for risky substance use. We propose a protocol to standardize the RT process for patients at high risk for developing severe SUD (WHO ASSIST score ≥ 27).

**Methods:** Meetings with stakeholders from two FQHCs in Los Angeles, CA were organized to develop a Screening Brief Intervention and Referral to Treatment protocol. Patients are screened using the WHO Alcohol, Smoking and Substance Involvement Screening Test (ASSIST). Scores of ≥ 27 are shared with behavioral health team, who further evaluate patients for SUD, refer to treatment, and complete a questionnaire to track if patients were assessed and connected to treatment.

**Results:** To date, 1638 participants have been screened. Of those, 3.7% (n = 61) were identified as having high-risk substance use. Average age was 42 years old. Patients reported a high-risk level of use on the following substances: alcohol (n = 29), cannabis (n = 14), sedatives (n = 9), methamphetamine (n = 10), prescription opioids (n = 4), cocaine (n = 3), prescription stimulants (n = 1), hallucinogens (n = 1). 14.8% reported currently using more than one substance. No participants had received SUD treatment in the past three months before the screening. Results were shared with the clinics according to the protocol.

**Conclusions:** These results and pilot RT protocol can support the integration of health screening to address SUD among patients at FQHCs. Next steps include continue to screen patients, monitor health records to investigate if patients connected to SUD services, if treatment was initiated, and barriers. Qualitative semi-structured interviews with patients, providers, and clinic stakeholders will be conducted to assess barriers and facilitators to implement a standardized RT protocol.

## NC-040 The prevalence of identified unhealthy alcohol use and alcohol use disorder (AUD) when alcohol screening is conducted online versus on paper

### Dane Patey^1^, Katherine Bradley^2^, Malia Oliver^2^, Theresa E. Matson^2^, Helen E. Jack^3^, Douglas Berger^4^, Tessa Steel^5^, Amy Lee^6^, Kevin Hallgren^7^

#### ^1^MedStAR Program, University of Washington, Seattle, WA, USA; ^2^Kaiser Permanente Washington Health Research Institute, Seattle WA, USA; ^3^Division of General Internal Medicine, Harborview Medical Center, University of Washington, Seattle WA, USA; ^4^General Medicine Service, Department of Veterans Affairs (VA) Puget Sound Health Care System, Seattle, WA, USA; ^5^Division of Pulmonary, Critical Care and Sleep Medicine, University of Washington, Seattle, WA, USA; ^6^Kaiser Permanente Washington, Mental Health and Wellbeing, Seattle, WA, USA; ^7^Behavioral Research in Technology & Engineering Center, Department of Psychiatry and Behavioral Sciences, UW Medical Center, Seattle, WA, USA

##### **Correspondence:** Katharine Bradley (katharine.a.bradley@kp.org)

*Addiction Science & Clinical Practice* 2023, **19(Suppl 1):**NC-040

**Background:** Little is known about how alcohol screening conducted via online patient portals in electronic health records (EHRs) compares to alcohol screening on paper in a clinic. Kaiser Permanente Washington began screening for unhealthy alcohol use with the AUDIT-C in 2015, using primarily paper forms. Starting in August 2020, patients were offered the option of pre-visit online AUDIT-C alcohol screening through the EHR patient portal.

**Materials and methods:** This study compared the results of AUDIT-C screening and diagnosis of alcohol use disorder (AUD) in adult patients completing online versus paper-based AUDIT-C screening from Sept 2020 to August 2021. Measures included the prevalence of (1) positive screens for unhealthy alcohol use (AUDIT-C ≥ 3 women; ≥ 4 men); (2) report of high-risk drinking (AUDIT-C 7–12); and (3) AUD diagnosis based on documentation of AUD ICD codes by a provider in the EHR.

**Results:** The study sample included 52,765 and 71,608 patients who completed online and paper-based screening, respectively. Among screened patients, online screening was associated with a higher prevalence of unhealthy alcohol use than paper-based screening (30.7% vs 26.7%), but a lower prevalence of high-risk drinking (2.1% vs 2.8%) and a lower prevalence of diagnosed AUD (0.8 vs 1.4%).

**Conclusion:** Alcohol screening via online patient portals may identify a higher proportion of patients with unhealthy alcohol use, potentially reaching some patients who do not routinely come into clinics for care. However, patient portals may identify a lower proportion of patients with high-risk drinking and AUD, which may have implications for AUD treatment. Further research is needed to understand whether these differences reflect (1) demographic or other population characteristics of those who complete screening online versus on paper in a clinic, (2) differences in measurement performance of online versus paper-based AUDIT-C screening, or (3) differences in providers’ use of screening information.

## NC-041 “Bebermenos (drinkless) 2.0”: virtual intervention to alcohol problems reduction

### Micaella Silva Leandro^1^, Keith Machado Soares^2^, Maria Lucia Oliveira de Souza Formigoni^3^

#### ^1^Graduate Program in Psychobiology, Federal University of Sao Paulo (UNIFESP) P, São Paulo, São Paulo, Brazil; ^2^Associação Fundo de Incentivo à PesquisaP, São Paulo, São Paulo, Brazil; ^3^Department of Psychobiology, Escola Paulista de Medicina, UNIFESP, São Paulo, São Paulo, Brazil

##### **Correspondence:** Maria Lucia Oliveira de Souza Formigoni (mlosformigoni@unifesp.br)

*Addiction Science & Clinical Practice* 2023, **19(Suppl 1):**NC-041

**Background:** In 2012, with the support of the World Health Organization, we developed a web-based intervention to reduce alcohol related problems and tested it in four countries (Brazil, Mexico, India and Belarus). The intervention “Drinkless” (called “Bebermenos v 1.0” in Brazil and Mexico) proved to be effective in reducing alcohol consumption, but the adherence to it was low (Schaub et al., 2021).

**Material and methods:** We analyzed the main criticisms and suggestions from users of that first version of the intervention in order to develop its second version.

**Results and conclusions:** The main criticisms pointed out by the users were: lack of a more intuitive guidance on the sequence of the activities proposed and better organization of the site; lack of an immediate feedback after the inclusion of data on consumption, advantages, disadvantages and drinking goals; lack of responsiveness on mobile devices (cell phones and tablets). Besides the inclusion of the suggested tools, in the current version we included others to collect data on the level of interaction of users with the application tools. New activities and resources were included to record the use of each activity, construction of a navigation script and guidance for users of the automated system, in addition to a script for use by a professional who could provide synchronous online guidance for users, with a summary of all activities performed. A randomized clinical trial is in progress to assess whether the new version of the site and the inclusion of guidance sessions on the use of the site mediated by a professional will improve the adherence to the program.

## NC-042 School-based SBIRT: program implementation data from 15 high schools

### Carolyn A. McCarty^1^, Erin Chase^2^, Margaret Soukup^3^, Delaney Knottnerus^3^, Evan Elkin^4^

#### ^1^Seattle Children’s Research Institute, ^2^University of Washington Department of Psychiatry and Behavioral Sciences, ^3^King County Department of Community and Human Services, ^4^Reclaiming Futures

##### **Correspondence:** Carolyn A. McCarty (cari.mccarty@seattlechildrens.org)

*Addiction Science & Clinical Practice* 2023, **19(Suppl 1):**NC-042

**Background:** Limited data are available on the implementation of Screening, Brief Intervention, and Referral to Treatment (SBIRT) in school settings. We sought to determine the prevalence of substance use and co-occurring mental health issues, and to characterize brief intervention and referral patterns based on our experience in implementing SBIRT across 15 high schools.

**Methods:** A total of 3462 high school students participated in electronic screening at their high schools in King County, Washington, between Jan 2022–July 2023, where they were administered the S2BI in addition to measures of mental health including depressive symptoms (PHQ-2), anxiety symptoms (GAD-2), bullying, self-harm, and suicidal ideation. Trained intervention staff then reached out to meet with students and provide brief motivational interviewing (BI), and documented the referrals and resources provided to students.

**Results:** Nearly one in five high school students (18%) reported some substance use during screening, with most common types being alcohol (15%), weed/marijuana (9.5%), e-cigarettes (7.1%), tobacco (3.6%), and other substances (2.9%). Over 57% of students using substances reported using more than one substance, and substance use frequently co-occurred with bullying (45%), anxiety symptoms (40%), depressive symptoms (31%), disordered eating habits (23%), self-harm (23%) or suicidal ideation (20%). BI was provided to 85% of students with substance use, and either referrals or resources were provided to 49% of those who received BI. The most common type of resource offered was websites (36%), and some students were also referred to the school counselor (28%) or in-school mental health (20%), and community-based counseling (21%).

**Conclusions:** The majority of students with substance use were willing to participate in brief intervention sessions with a trained interventionist at school following screening. The high co-occurrence rate of substance use and mental health issues highlights the importance of broad attention to the psychosocial needs of high school students as part of the SBIRT process.

## NC-043 Adaptation in French of the tobacco, alcohol, prescription medication and other substance (TAPS) tool: a new multi-substance screening use for French-speaking primary care patients

### Angéline Adam^1^, Céline Gachoud^1^, Charlotte Eidenbenz^2^, Christine Cohidon^3^, Nicolas Senn^3^, Isabelle Jacot-Sadowski^4^, Jean-Bernard Daeppen^1^, Jennifer McNeely^5^, Nicolas Bertholet^1^

#### ^1^Department of psychiatry, Addiction Medicine, Lausanne University Hospital and University of Lausanne, Lausanne, Switzerland; ^2^Translation Eidenbenz, La Tour-de-Peilz, Switzerland; ^3^Department of Family Medicine, Center for Primary Care and Public Health (Unisanté), University of Lausanne, Lausanne, Switzerland; ^4^Department of Health Promotion and Prevention, Center for Primary Care and Public Health (Unisanté), University of Lausanne, Lausanne, Switzerland; ^5^New York University Grossman School of Medicine, New York, NY, USA

##### **Correspondence:** Angéline Adam (angeline.adam@chuv.ch)

*Addiction Science & Clinical Practice* 2023, **19(Suppl 1):**NC-043

**Background:** Screening for substance use is recommended in primary care but underdone. A short and self-administered tool could facilitate screening, but it is not available in French. We translated and adapted the TAPS into French. TAPS is a 13-item screening and assessment for tobacco, alcohol, prescription and other drug use.

**Materials and methods:** We used a two-steps procedure. (1) A reconciled version was obtained from two forward translations (English to French), back-translated into English, repeated until a consensus was reached. Translations were done by four independent professional translators. (2) Then two consecutive qualitative assessments were conducted among French speaking primary care adult patients providing feedback in one-to-one semi-structured interviews.

**Results**: For 10/13 items, consensus was reached after one sequence of translation and back-translation. A second sequence was needed for three items. Mean (SD) age of participants was 58 (19); 35% were female. Content analysis: in the first round of interviews (n = 10), seven participants reported that the alcohol and prescription drug items had too many numerical indications (e.g. *In the past 12 months, how often have you had five or more drinks containing alcohol in one day?*), were too long, and required several reads to be understood. Participants reported confusion between usual prescription and misuse of drugs. As a result, we modified the formatting of the items and used more popular wording. In the second round of interviews (n = 7), all participants still reported difficulties with the alcohol item with a need to process complex information combining numerical thresholds, frequency, and time. One participant reported semantic challenges with the prescription drug item.

**Conclusions:** When adapting the TAPS into French, comprehension challenges related to the meaning of prescription drug use and complex numerical indications for the alcohol item were identified. Specifically, the alcohol screening question is particularly challenging and requires complex thinking.

## NC-044 Lessons from two pilot randomized controlled trials that systematically developed and tested contextually relevant text-messaging brief interventions for tobacco cessation and hazardous drinking in India

### Miriam Sequeira^1^, Richard Velleman^1,2^, Abhijit Nadkarni^1,3^

#### ^1^Addictions and related-Research Group, Sangath, Goa, India; ^2^University of Bath, Bath, UK; ^3^Department of Population Health, London School of Hygiene and Tropical Medicine, London, UK

##### **Correspondence:** Miriam Sequeira (miriam.sequeira@sangath.in)

*Addiction Science & Clinical Practice* 2023, **19(Suppl 1):**NC-044

**Background:** In India the treatment gap for tobacco use and alcohol use disorders (AUDs) is 92% and 86% respectively. Digital interventions have been touted as an innovative cost-effective solution. However, most of these digital interventions are developed in high-income countries (HICs). This paper compares the processes and outputs of two studies—ToQuit and AMBIT—which systematically developed contextually relevant mobile phone–delivered brief interventions for tobacco use and hazardous drinking respectively.

**Methods**: Mixed methods studies following five steps: (1) Extraction of intervention components from the most recent and relevant high quality systematic reviews; (2) In-depth interviews (IDIs) with alcohol and tobacco users and Indian experts in the field; (3) Surveys with international experts to rate each intervention component on the feasibility, acceptability and perceived effectiveness when delivered via text messages; (4) Intervention development workshops with local stakeholders; and (5) Feasibility RCT to test the intervention.

**Results**: After testing bidirectional messaging and Interactive Voice Response (IVR) in addition to text message delivery, the final version of both interventions included unidirectional text-messages delivered over 8 weeks on days of the week tailored to the participant’s preference. The AMBIT study also tested content delivered via Interactive Voice Response (IVR). The ToQuit intervention employed the Behaviour Change Taxonomy framework and included 27 behaviour change techniques (BCTs) like goal setting, avoidance of cues to behaviour, distraction, substitution among others. The AMBIT intervention included contextually relevant messages on safe drinking limits, personalized feedback, alcohol reduction strategies among others. More than 85% of participants completed the intervention in both studies and positive but insignificant changes were found in treatment outcomes in the intervention arm compared to the control arm.

**Conclusions:** Although our studies fared well on acceptability and feasibility, developing a digital intervention that is also equally effective requires multiple rounds of testing with local stakeholders.

## NC-045 Why me? If I just wanted to have fun. Piloting a strategy of early identification and brief intervention to prevent and manage alcohol-related problems in underage population at risk

### Carla Bruguera^1^, Carme Castells^2^, Núria Tomàs^2^, Montse Navarra^3^, Pol Bruguera^4^, Lidia Martínez^5^, Griselda Esquerra^6^, Eulàlia Sot^1^, Estela Diaz^7^, Lidia Segura^1^, Joan Colom^1^

#### ^1^Subdirectorate General on Addictions, HIV, STD and Viral Hepatitis, Public Health Agency of Catalonia. Barcelona, Catalonia, Spain; ^2^Centre d’atenció primaria de La Pobla de Segur. La Pobla de Segur, Catalonia, Spain; ^3^Sistema d’emergències mèdiques, Alt Pirineu i Aran division. Tremp, Catalonia, Spain; ^4^Unitat de Conductes Addictives, Hospital Clínic. Health and Addictions Research Group, IDIBAPS. Barcelona, Catalonia, Spain; ^5^Sant Joan de Déu Hospital, Barcelona, Catalonia, Spain; ^6^Catalan Health Service, Alt Pirineu i Aran division. Tremp, Catalonia, Spain; ^7^Catalan Health Service, Barcelona, Catalonia, Spain

##### **Correspondence:** Carla Bruguera (cbrugueras@gencat.cat)

*Addiction Science & Clinical Practice* 2023, **19(Suppl 1):**NC-045

**Introduction:** Adolescence represents an important period in the drinking onset, estimated to be on average at 13.9 years old in Catalonia. In the context of the Beveu Menys (Drink less) program we are piloting a strategy to prevent and manage alcohol-related problems in underage population. A secondary aim is to validate the NIAAA screening tool for underage population in our context.

**Methods:** The project is being deployed in all health centres of one region in Catalonia for at least six months. The strategy includes: (1) screening and brief interventions (BI) to population from 12 to 18 years old during Primary health care (PHC) visits; (2) BI and referral to PHC for follow up of minors attended in emergency rooms and ambulances due to alcohol intoxication; (3) follow-up of minors at risk transitioning from paediatric to adult PHC. Implementation components include: (1) organizational actions such as inclusion of screening tools in the medical records (2) actions at professional level such as protocols and training and (3) actions at population level such as informational leaflets.

**Results:** 18 referents have been identified (at least one in each centre) to coordinate the deployment in their service and 77 professionals have been trained. 74.1% of participants worked in PHC and and 49.5% were nurses. 70.2% professionals had previously received less than 5 h of training on the topic, almost half of the sample reported asking about alcohol rarely although a vast majority considered important discussing alcohol with young people. Implementation is ongoing now. Preliminary results of changes and impact of the intervention will be presented during the conference.

**Conclusion:** Mobilization of a health region towards the implementation of an integrated strategy to prevent and manage alcohol-related problems in underage population is feasible and service providers and professionals consider it very important.

## NC-046 Development of an optimized opioid misuse prevention program for at-risk employees

### Stephen Hebard^1^, William B. Hansen^1^, GracieLee M. Weaver^1^, Jeff Milroy^1,2^, David Wyrick^1,2^, Kelly Rulison^1^, Scarlett Ruppert^2^

#### ^1^Prevention Strategies, Greensboro, NC, USA; ^2^Department of Public Health Education, UNC Greensboro, Greensboro, NC, USA

##### **Correspondence:** Scarlett Ruppert (sbruppert@uncg.edu)

*Addiction Science & Clinical Practice* 2023, **19(Suppl 1):**NC-046

**Background:** Employees in industries with high rates of opioid dispensing and non-fatal work-related injury (construction, healthcare, etc.) are at increased risk for opioid misuse. Workplace costs associated with opioid misuse is estimated at $25.6 billion due to lost productivity, turnover, and premature death of employees. The growing presence of the opioid crisis in the workplace brings to light the need for more evidence-based interventions to prevent opioid misuse among at-risk employees. Our team used a multiphase optimization strategy (MOST) to develop, test, and optimize an opioid misuse prevention program tailored for workers in high-risk industries.

**Materials and methods:** Using the Preparation Phase of the MOST framework, our team conducted literature reviews, interviews, and focus groups to develop a conceptual model for opioid misuse. An online prevention program based on our conceptual model was pilot tested with healthcare workers. Results from the Preparation Phase were used to inform the Optimization and Evaluation Phases of the MOST framework. Using a factorial design, two optimization trials will evaluate and strengthen the intervention components.

**Results:** Survey results from 856 healthcare workers during the preparation phase supported our conceptual model, showing significant effects of knowledge, beliefs, attitudes, social norms, perceived behavioral control, and patient-provider communication on intentions to misuse prescription opioids. Preparation phase results informed further development of program modules to be tested with factorial design across healthcare and construction industries. Phase II, consisting of the optimization and evaluation phases, is ongoing with results expected before presentation in September.

**Conclusions:** This research addresses a critical need for intervention to prevent opioid misuse among at-risk workers. This intervention is built with a primary prevention lens and undergoing optimization trials that will inform a final product that is more efficient and effective.

## NC-054 Disparities in alcohol assessment for primary care patients with alcohol-related health conditions

### Katherine J. Karriker-Jaffe^1^, Yachen Zhu^2^, Yu Ye^2^, Joanne Delk^2^, Aryn Z. Phillips^3^, Kara M. K. Bensley^2^, Nina Mulia^2^

#### ^1^RTI International, Berkeley, CA USA; ^2^Public Health Institute’s Alcohol Research Group, Emeryville, CA USA; ^3^University of Maryland School of Public Health, College Park, MD USA

##### **Correspondence:** Kate Karriker-Jaffe (kkarrikerjaffe@rti.org)

*Addiction Science & Clinical Practice* 2023, **19(Suppl 1):**NC-054

**Background:** Cost-efficient, primary care-based alcohol screening of people with chronic health conditions is important for improving population health. This study assessed disparities in alcohol assessment among this at-risk patient population.

**Materials and methods:** Data are from the 2013–2019 National Surveys on Drug Use and Health. The analytic sample was restricted to respondents with 1+ alcohol-related chronic condition who also had at least one primary care visit, but no ER visits or hospital stays, in the past year (N = 46,014). Past-year receipt of primary care-based alcohol assessment was indicated by respondent self-reports that a healthcare provider inquired about whether, how often, or how much they drink or about alcohol-related problems. Logistic regression assessed disparities in alcohol assessment, with sequential models adjusting for demographics, insurance coverage, socioeconomic status (SES), alcohol-related health conditions, and past-year primary care visits.

**Results**: There were robust disparities in primary-care based alcohol assessment. Even when accounting for differences in insurance status, number of primary care visits, number of alcohol-related health conditions, and survey year, several patient groups were significantly less likely to receive alcohol screening: people who identified as Black or Asian/Pacific Islander (vs. White), people with lower SES (less than 4-year college degree; lower income; and/or unemployed), patients from rural (vs. urban) areas, and older (50–64, 65+ vs. 18–25) patients. The most striking disparities were found for the oldest patients (AOR = 0.37 for adults 65+), Asian/Pacific Islanders (AOR = 0.45), and those with less than a high school degree (AOR = 0.47) in the overall sample, as well as in specific subgroups of patients with hypertension, diabetes, and/or heart conditions (in the latter, Black patients also had lower odds of screening: AOR = 0.60).

**Conclusions**: Robust disparities in alcohol assessment for primary care patients with alcohol-related chronic conditions underscore urgent need for interventions to rectify these missed opportunities and advance equity in primary care screenings.

## NC-055 Cannabis use among patients in primary care: findings from a screening program implemented in a large urban health system

### Lillian Gelberg^1,2^, Marjan Javanbakht^3^, Julia Koerber^3^, Whitney N. Akabike^1^, Lawrence Dardick^4^, Clara Lin^4^, Steve Shoptaw^1^

#### ^1^UCLA David Geffen School of Medicine, Department of Family Medicine, Los Angeles, CA, USA; ^2^UCLA Fielding School of Public Health, Department of Health Policy and Management, Los Angeles, CA, USA; ^3^UCLA Fielding School of Public Health, Department of Epidemiology, Los Angeles, CA, USA; ^4^UCLA David Geffen School of Medicine, Department of Internal Medicine, Los Angeles, CA, USA

##### **Correspondence:** Lillian Gelberg (lgelberg@mednet.ucla.edu)

*Addiction Science & Clinical Practice* 2023, **19(Suppl 1):**NC-055

**Background:** In light of cannabis use legalization, most healthcare systems do not have a standardized way of collecting information on patients’ cannabis use, even though cannabis use could impact health outcomes. We implemented automated, computerized, patient self-administered screening for cannabis use among patients of a large urban academic health system.

**Materials and methods:** The Tobacco and Cannabis Questionnaire (based on the WHO ASSIST) was sent out via the electronic health record (EHR) patient portal to primary care patients 18+ years.

**Results:** From 2021–2022, 113,229 patients completed cannabis screening: median age 47 years, 59% female, and White/Caucasians comprised the single largest racial/ethnic group (36%). Recent cannabis use (past 3 months) was reported by 14%, and was more common among young adults 18–29 years vs. older adults 60+ years (25% vs 7%); men vs women (17% vs. 12%). Among cannabis users, the most common modes of use were inhalation (66%) and ingestion (65%), with 44% using multiple modes; 34% had ASSIST scores indicative of moderate—to high-risk for cannabis use disorder (CUD). Nearly equal numbers used cannabis for medical reasons vs. strictly non-medical reasons (49% vs 51%), with those using it for medical reasons being more likely to be older than 18–29 years and living in the most disadvantaged neighborhoods. Among all cannabis users, 78% used cannabis to manage symptoms especially for mental health symptoms (61%) and sleep (60%), followed by pain (41%). Interestingly, among patients who reported using cannabis for non-medical reasons only, 60% reported symptoms for which they used cannabis.

**Conclusions:** This study is among the first to implement a computerized, self-administered screener as part of the EHR in a large health system. Cannabis use rates are high, with patients using multiple modes and high frequency of risky cannabis use levels, suggesting the importance of screening in primary care.

## NC-057 Estimating adolescent drinking trajectories in the United States: a three-step approach

### Md Zubab Ibne Moid

#### UNC Greensboro, Greensboro, NC, USA

##### **Correspondence:** Md Zubab Ibne Moid (m_moid@uncg.edu)

*Addiction Science & Clinical Practice* 2023, **19(Suppl 1):**NC-057

**Background:** Understanding how adolescents transition over time across levels of drinking is crucial when trying to understand the lifetime effects of adolescent alcohol brief interventions. This study estimates the transition probabilities between different levels of alcohol consumption to understand the drinking trajectories of adolescents in the United States.

**Materials and methods:** Using Bayesian Simulation techniques, we computed transition probabilities for adolescents in the United States, specifically focusing on the four drinking risk levels defined by the World Health Organization (low, medium, high, and very high risk), as well as abstinence and alcohol dependence. We used the National Longitudinal Survey of Youth 1997 cohort (NLSY97) and the National Survey on Drug Use and Health (NSDUH) datasets to calculate these transition probabilities. We validated the transition probabilities using an NSDUH cohort which was not used to calculate transition probabilities.

**Results:** We find that transition probabilities are significantly different for age cohorts and males versus females. We find that the likelihood of progressing to alcohol dependence is substantially greater for adolescents that engage in heavy drinking at younger ages.

**Conclusions:** No previous research has attempted to calculate transition probabilities between different levels of alcohol consumption of adolescents in the United States using national-level data. These transition probabilities are essential to understanding the progression of alcohol use over a lifetime and can be used to estimate the long-term cost-effectiveness of alcohol brief interventions for adolescents.

## NC-058 Screening, brief intervention, and referral to treatment (SBIRT) cascade of care and correlates: variation by alcohol use state

### Carolina Barbosa^1^, Joanne Delk^2^, William Dowd^1^, Jessica Duncan Cance^1^, Ivette Rodriguez Borja^1^, Nina Mulia^2^, Katherine J. Karriker-Jaffe^1^

#### ^1^RTI International, Research Triangle Park, NC, USA; ^2^Public Health Institute’s Alcohol Research Group, Emeryville, CA, USA

##### **Correspondence:** Carolina Barbosa (cbarbosa@rti.org)

*Addiction Science & Clinical Practice* 2023, **19(Suppl 1):**NC-058

**Background:** SBIRT can identify and reduce harmful drinking, thereby reducing the risk of alcohol use disorder (AUD).

**Materials and methods:** Using 2021 National Survey on Drug Use and Health data, we assessed the weighted proportion of adults (n = 47,291) who reported, for the past 12 months: (1) utilizing healthcare; (2) being screened about alcohol use; (3) receiving a brief intervention (BI) for alcohol misuse; (4) being referred to alcohol treatment; and (5) receiving alcohol treatment. The SBIRT care cascade was assessed across seven alcohol use states: abstinent, drinker, risky drinker, 1 AUD symptom, and mild, moderate, and severe AUD. Multivariable logistic regression models assessed the likelihood of receiving each step by socio-demographic characteristics.

**Results:** Past-year AUD prevalence was 11.4%. About 80% of adults utilized healthcare in the previous year, of which 69% reported being screened for alcohol use. Individuals with severe AUD had the highest screening rates (89%), followed by moderate (87%) or mild (82%). Only 8% of risky drinkers or people with one or more AUD symptoms reported receiving BI, with higher rates among severe (30%), moderate (18%), or mild (9%). Prevalence of being referred and receiving treatment increased with AUD severity. Despite higher likelihood of receiving health care than males, females were less likely to receive BI, be referred to treatment, or receive treatment. Compared to White individuals, Hispanic individuals were less likely to use healthcare, and those with severe AUD were less likely to receive BI and treatment referral. Compared to White individuals, Black risky drinkers were less likely to use healthcare and to be screened for alcohol use.

**Conclusions:** Despite wide healthcare utilization and screening, there are substantial gaps in receiving the remaining steps of the SBIRT cascade. Gender and racial disparities in receiving SBIRT need to be addressed, particularly among risky drinkers and people with AUD.

## NC-059 Community pharmacy-provided injectable naltrexone—a year in the life of a resource-development project

### James H. Ford II, Aaron Gilson, Michele Gassman

#### University of Wisconsin School of Pharmacy–Social and Administrative Sciences Division, Madison, Wisconsin, USA

##### **Correspondence:** James H Ford II (jhfordii@wisc.edu)

*Addiction Science & Clinical Practice* 2023, **19(Suppl 1):**NC-059

**Background:** Opioid use disorder (OUD) is a major public health issue in the United States. The preferred treatment approach, which combines the use of approved medications for OUD (MOUD) with counseling and behavioral therapies, represents an evidence-based approach to treat individuals living with an OUD. However, access to MOUD has not kept up with increased demand and new approaches are needed. In Wisconsin, pharmacists can provide long-acting injectable naltrexone, an approved and effective MOUD treatment.

**Materials and methods:** We gathered information to identify injectable naltrexone best practices and tools for community pharmacies when initiating or promoting an injectable naltrexone service to the community (including drug courts), potential provider partners, and clinics. Information obtained for this resource-development project was generated through a systematic process of comprehensive literature reviews, semi-structured interviews and focus groups with key stakeholders (e.g., pharmacists, nurses, and physicians), surveys, and expert review and validation, to ensure completeness and representativeness of the educational content. Study was approved by the UW-Madison Minimal Risk IRB (2021–0217).

**Results:** Data from statewide surveys (n = 88) suggest that pharmacists’ roles in providing MOUDs, including injectable naltrexone, are expanding. Focus groups (n = 5) and interviews (n = 15) identified that community pharmacy provided MOUD creates a positive impact through greater flexibility and accessibility, especially in rural areas. The focus groups and interviews also informed the development of community pharmacists resources highlighting the benefits of community pharmacists’ injectable naltrexone for patients, pharmacists, providers, and treatment court coordinators.

**Conclusions:** This research addresses national calls for increased pharmacists’ involvement to expand treatment access for OUD and alcohol use disorder patients. Direct stakeholder engagement informed the development of these outreach resources. Utilization could help expand access to long-acting injectable medications by establishing community pharmacist partnerships. In turn, it broadens available treatment networks and brings research into practice by leveraging existing policies and regulations.

## NC-060 A scoping review of healthcare workers’ perspectives of outpatient provision of methadone

### James H. Ford II, Sara Hernandez, Aaron Gilson, Michele Gassman, Te-Lien Ku

#### University of Wisconsin School of Pharmacy–Social and Administrative Sciences Division, Madison, Wisconsin, USA

##### **Correspondence:** James H Ford II (jhfordii@wisc.edu)

*Addiction Science & Clinical Practice* 2023, **19(Suppl 1):**NC-060

**Background/objectives:** A recent Call To Action focused on expanding methadone treatment access for individuals with OUD. One priority was optimal educational and support structures, including training to provide methadone across healthcare settings (e.g., primary care, opioid treatment programs (OTPs), pharmacies) and healthcare workers (e.g., providers, pharmacists). This scoping review addresses this gap.

**Methods:** PubMed, APA PsycInfo, CINAHL, and Web of Science databases were searched for English language articles (published 2010 2022) containing evidence of healthcare worker’s knowledge, attitudes, and methadone stigma related to providing methadone in the United States outpatient setting. The scoping review focused on two staff perspectives–healthcare workers generally and pharmacists specifically.

**Results:** A total of 2747 articles were identified; 15 ultimately met inclusion criteria. Settings included: substance use programs (n = 7), primary care or provider offices (n = 3), OTPs (n = 3), and pharmacies (n = 2). All articles examined methadone related attitudes. While methadone is the oldest MOUD, a majority of studies (n = 10) illustrated continued methadone-related stigma held by healthcare workers. Importantly, following COVID related policy changes authorizing more take home doses of methadone, OTP clinicians expressed varied attitudes about whether increased access created greater patient risk and program liability from diversion and overdose. One of the two articles assessing knowledge suggested that, even when a majority of pharmacists correctly answered knowledge questions, a significant minority had misunderstandings that could undermine effective treatment.

**Conclusions:** Given the current imbalance between MOUD treatment demand and availability, expanding outpatient provided methadone is necessary. Pharmacists represent a critical but underutilized MOUD access point through partnering with OTP providers to distribute methadone. For this to occur efficiently, the scoping review identified potential areas to improve knowledge and attitudes. With optimal methadone educational and support structures in place, research can explore the impact of different implementation strategies on methadone expansion, quality of care, effectiveness, safety, and stigma.

## NC-061 Effectiveness of a brief negotiational intervention to reduce harmful and hazardous alcohol use in the emergency department: a pragmatic randomized adaptive clinical trial in Moshi, Tanzania

### Catherine A. Staton^1,2,4^, Linda Minja^3,4^, Joao Vitor Perez de Souza^2,4^, John A. Gallis^1^, Pollyana Coelho Pessoa Santos^4^, Mia Buono^1,4^, Francis Sakita^3^, Judith Boshe^3^, Ashley Joyce Phillips^1,2,4^, Joao Ricardo Nickenig Vissoci^1,2,4^, Blandina T. Mmbaga^3,4,5^

#### ^1^Duke Global Health Institute, Duke University, Durham, NC, USA; ^2^Department of Emergency Medicine, Duke University School of Medicine, Durham, NC, USA; ^3^Kilimanjaro Christian Medical Centre, Moshi, Tanzania; ^4^Global Emergency Medicine Innovation and & Implementation Research Center, Duke University, Durham, NC, USA; ^5^Kilimanjaro Clinical Research Institute, Moshi, Tanzania

##### **Correspondence:** Catherine A. Staton (catherine.lynch@duke.edu)

*Addiction Science & Clinical Practice* 2023, **19(Suppl 1):**NC-061

**Background:** Alcohol use disorder (AUD) is a major health challenge in Tanzania, with systemic and human resource challenges making effective care difficult to access. This study aims to evaluate the potential of a novel approach to tackling this issue: a culturally adapted brief negotiation interview (BNI), combined with SMS boosters. We report the reduction in binge drinking behavior among patients at 3 months post-hospital discharge from Kilimanjaro Christian Medical Centre (KCMC) in Moshi, Tanzania.

**Methods:** This randomized controlled trial enrolled 449 adult injury patients admitted to KCMC who used alcohol prior to injury or screened positive for AUD. Participants who were randomized in the intervention group received the culturally adapted BNI, “Punguza Pombe Kwa Afya Yako” plus weekly text messages encouraging alcohol reduction. This hybrid approach of face-to-face BNI and digital reminders aimed at enhancing the retention of the intervention. Mixed effects zero inflated negative binomial models were used to assess intervention effects.

**Results:** An interim analysis was conducted after 336 patients completed 3 months follow-up (228 intervention, 108 control). Both groups showed reduction in binge drinking days, while the intervention group had a significant reduction in binge drinking days compared to the usual care group (predicted mean difference between groups: 1.3 days, [CI 1.22–1.38] days, p = 0.01529), indicating that the intervention was effective. There were also significant reductions in quantity and frequency of alcohol use in favor of the intervention group.

**Conclusions:** Our findings strongly suggest that the culturally adapted BNI, enhanced with SMS booster, serves as an effective intervention to mitigate harmful alcohol use behavior in Tanzania. It is also possible that the BNI method + SMS booster holds potential for addressing AUD effectively. Further in-depth research is essential to fully explore the long-term impact of this intervention, evaluate its scalability, and refine its implementation for wider application.

## NC-062 Using the ADAPT guidance to cultural adapt a brief intervention to reduce alcohol use among injury patients in Tanzania

### Catherine A. Staton^1,2^, Joao Vitor Perez de Souza^2^, Armand Zimmerman^1^, Msafiri Pesambili^3^, Ashley J. Phillips^1,2^, Anna Tupetz^2^, Judith Boshe^3^, Michael H. Pantalon^5^, Monica Swahn^6^, Blandina T. Mmbaga^3,4^, Joao Ricardo Nickenig Vissoci^1,2^

#### ^1^Duke Global Health Institute, Duke University, Durham, NC, USA; ^2^Department of Emergency Medicine, Duke University School of Medicine, Durham, NC, USA; ^3^Kilimanjaro Christian Medical Centre, Moshi, Tanzania; ^4^Kilimanjaro Clinical Research Institute, Moshi, Tanzania; ^5^Department of Emergency Medicine, Yale University; ^6^Department of Population Health Sciences, School of Public Health, Georgia State University, Georgia USA; ^7^Department of Emergency Medicine, University of Maryland, Baltimore, MD USA

##### **Correspondence:** Catherine A. Staton (catherine.lynch@duke.edu)

*Addiction Science & Clinical Practice* 2023, **19(Suppl 1):**NC-062

**Background:** Harmful alcohol use is a leading risk factor for injury-related death and disability in low- and middle-income countries (LMICs). Brief negotiational interventions (BNIs) administered in emergency departments (EDs) to injury patients with alcohol use disorders (AUDs) are effective in reducing post-hospital alcohol intake and re-injury rates. The effectiveness of BNIs in LMICs remains largely unknown. Given the high prevalence of alcohol-related injury in the Kilimanjaro region of Tanzania, we culturally adapted a BNI to reduce post-injury alcohol use for implementation in this patient population.

**Methods:** Following the ADAPT guidance, we used an iterative, multiphase process to culturally adapt a high-income country BNI to the Tanzanian context. Our team consisted of local healthcare professionals and international academic and clinical professionals to integrate our extensive mixed-methods patient data to adapt this intervention. Design group discussions were used to discuss research results, interpret findings, discuss the goals of the intervention, identify and suggest areas of adaptation of the intervention and specific adaptations to the BNI protocol. Objective assessments of our BNI protocol as well as a BNI assessment scale was developed to guide intervention fidelity.

**Results:** We designed the Punguza Pombe Kwa Afya Yako (PPKAY); a one-time, 15-min nurse-led BNI promoting safe alcohol use and behavior change. Alterations to the protocol included adjustments to greeting, alcohol discussion initiation, harmful use notification, graphics visualization, motivation tactics, and and which behavior changes are encouraged. We also created an accompanying BNI Assessment Scale which evaluates the adherences to the protocol and motivational interviewing tenants.

**Conclusions:** The PPKAY intervention is the first alcohol BNI which was culturally adapted for delivery to injury patients in an African ED. Our study lays a framework and method for other low resourced settings to integrate cultural adaptation into the implementation of a BNI in low resource EDs.

## NC-063 Translation and adaptation of the brief negotiation interview adherence scale for the Tanzanian culture

### Jiawen Wu^1,2^, Ashley J. Phillips^2,3^, Thiago Augusto Hernandes Rocha^2,3^, Joao Ricardo Nickenig Vissoci^1,2,3^, Judith Boshe^2,4,5^, Blandina Mmbaga^1,2,4,5^, Vanessa Menezes Menegassi^2^, Natan Nascimento de Oliveira^2^, João Vítor Perez de Souza^2,3^, Catherine A. Staton^1,2,3^

#### ^1^Duke Global Health Institute, Duke University, Durham, NC, USA; ^2^Global Emergency Medicine Innovation and & Implementation Research Center, Duke University, Durham, NC, USA; ^3^Department of Emergency Medicine, Duke University School of Medicine, Durham, NC, USA; ^4^Kilimanjaro Christian Medical Centre, Moshi, Tanzania; ^5^Kilimanjaro Clinical Research Institute, Moshi, Tanzania

##### **Correspondence:** Catherine A. Staton (catherine.lynch@duke.edu)

*Addiction Science & Clinical Practice* 2023, **19(Suppl 1):**NC-063

**Background:** Harmful or hazardous alcohol use is a grave public health problem, causing over 3.3 million deaths annually worldwide. Brief Negotiation Interview (BNI) can help reduce alcohol use and modify drinking patterns. Considering this, the present study’s objectives were to (1) develop a Swahili version of the BNI Adherence Scale (BAS) adapted to the Tanzanian culture; (2) support the previously published culturally adapted BNI “Reduce Alcohol for Your Health” a nurse-delivered intervention (PPKAY); and, (3) analyze the psychometric properties of the BAS scale such as internal consistency and factor structure.

**Methods:** This was a cross-sectional evaluation of the adherence of emergency department health care practitioners to the BNI components among patients with harmful alcohol use presenting for care at the Kilimanjaro Christian Medical Centre in Moshi, Tanzania. The translation and cross-cultural adaptation involved the ‘back-translation’ method. Five research nurses, which evaluated up to twelve BNI sessions, completed 108 individual assessments. The validity related to the internal structure of the different models was evaluated by Confirmatory Factor Analysis. The internal consistency was measured by the Cronbach’s alpha, McDonald’s Omega coefficient and Composite Reliability.

**Results:** Both 2-factor and 3-factor models presented good internal consistency and factor loadings. High values were found for TLI, CFI and NNFI (> 0.90). Both RMSEA values were smaller than 0.08. The 3-factor model could explain 77%, 65% and 65% variables’ variance while the 2-factor model could explain 76% and 53% variables’ variance of each dimension.

**Conclusions:** This is the first study to translate and adapt the BNI Adherence Scale to Tanzanian culture. The 3-factor model performed better with good fit indices. The adapted scale showed to be a reliable instrument to assess healthcare providers’ adherence to BNI and is a supportive tool and important component of the previously culturally adapted BNI PPKAY.

## NC-064 E-learning educational resource to improve brief intervention in smoking cessation skills among healthcare undergraduate students

### Tereza Barroso^1^, Idoia Pardavila^2^, Sandra Tricas-Sauras^3^, María Duaso^4^, Jordi Vilaplana^5^, Carmen Moreno^6^, Cristina Martínez^7^

#### ^1^Health Sciences Research Unit: Nursing, Nursing School of Coimbra, Coimbra, Portugal; ^2^Universidad de Navarra, Pamplona, Spain; ^3^Université Libre de Bruxelles (ULB), Brussels, Belgium; ^4^King’s College London, London, UK; ^5^Universitat de Lleida, Lerida, Spain; ^6^Universitat de Barcelona, Barcelona, Spain; ^7^Institut Català d’Oncologia, L’Hospitalet, Barcelona, Spain

##### **Correspondence:** Tereza Barroso (tbarroso@esenfc.pt)

*Addiction Science & Clinical Practice* 2023, **19(Suppl 1):**NC-064

**Background/objectives:** The lack of smoking cessation training for healthcare professionals during their academic years negatively impacts the quality of care provided. Thus, developing and implementing training programmes that enhance the brief intervention in smoking cessation skills among HealthCare undergraduate students is essential.

**Aim:** To evaluate the effectiveness of an online training programme to develop brief intervention in smoking cessation skills among HealthCare undergraduate students.

**Methods:** Pre-post-test study involving 851 students from seven universities across Spain (n = 663), the United Kingdom (n = 124), Belgium (n = 23), and Portugal (n = 41). The online training programme consisted of five theoretical modules, five videos, and three virtual simulation cases and lasted an equivalent of 30 learning hours. It was conducted between January 2020 and June 2022. Smoking cessation competency was achieved if students scored greater than 5 (out of 10) on the knowledge test and overall simulation score. The knowledge acquisition was evaluated through a multiple-choice test, and the attitudes and abilities were assessed through a simulation algorithm, both developed by experts in education and smoking cessation.

**Results:** Overall, 86% of participants achieved smoking cessation competency. Students’ knowledge significantly increased by 3.5 points, from a mean pre-programme score of 3.79 to a mean post-program score of 7.33 (p < 0.01). Moreover, they obtained a mean score of 7.5 out of 10 for attitudes and skills, which is more than satisfactory.

**Conclusions:** This innovative online training programme increased smoking cessation competence compared with the baseline situation among health profession students in four European countries. INSTrUCT is available free of cost to all the scholars and universities that want to use it: https://instruct-elearning.eu/.

## Posters

## Best Poster: NC-P05 Changes in prevalence of substance use among patients of community health centers in Los Angeles over time: comparative analysis from 2013 to 2023

### Cristina Batarse^1^, Quynh T. N. Vo^1^, Daisy Hernandez-Casas^1^, Melvin W. Rico^1,4^, Stephanie Sumstine^2^, Leticia Cazares^1^, Amanda Sirisoma^1^, Maria Vasquez^1^, Jack Tsai^5^, Armen Arshakyan^6^, Lillian Gelberg^1,3^, Dallas Swendeman^2^

#### ^1^Department of Family Medicine, David Geffen School of Medicine at UCLA, Los Angeles, California, USA; ^2^Department of Psychiatry and Biobehavioral Sciences, David Geffen School of Medicine at UCLA, Los Angeles, California, USA; ^3^Department of Health Policy & Management, Fielding School of Public Health, UCLA, Los Angeles, California, USA; ^4^Charles R. Drew University of Medicine and Science, Los Angeles, California, USA; ^5^The Children's Clinic, “Serving Children and Their Families” Long Beach, California, USA; ^6^Saban Community Clinic, Los Angeles, California, United States

##### **Correspondence:** Cristina Batarse (cristinabatarse@gmail.com)

*Addiction Science & Clinical Practice* 2023, **19(Suppl 1):**NC-P05

**Background:** In 2019, 16.9% of Californians reported using a substance in the past month. Little is known about the prevalence of drug use in FQHCs, and there are no comparative studies of rates of drug use in primary care (PC) settings across time. We examined changes in the prevalence of substance use over time among FQHCs in LA.

**Methods:** Data from the NIDA-funded Quit Using Drugs Intervention Trial (QUIT)-Mobile screening and brief intervention for risky drug use in adult patients of FQHCs was used to compare drug use prevalence to the 2013 QUIT-Binational study that also recruited PC patients from FQHCs in LA (N = 2507). The WHO Alcohol, Smoking, and Substance Involvement Screening Test (ASSIST) was used to screen patients for substance use. Problem drug use (Moderate-to-High) is defined as ASSIST 4+ score for at least one drug.

**Results:** A total of 1638 were screened as of March 2023. The prevalence of moderate-to-high drug use was higher in March 2023 (27.5%) compared to 2013 (19.4%). Problem cannabis use increased from 12.5 to 19.4%, as did problem alcohol use from 15.2 to 37.1%. Problem prescription sedative use increased from 4.6 to 5.4%. Problem tobacco use decreased from 20.5 to 17.6%, as did problem prescription opioid use from 4.6 to 3.7%. Problem stimulant use decreased from 7.3 to 5.8%. There were no changes in prevalence use of hallucinogens.

**Conclusions:** The increase in problem cannabis use may reflect post-legalization of recreational cannabis factors. Problem alcohol use alarmingly more than doubled, possibly influenced by COVID-19 pandemic-associated alcohol consumption. PC settings are critical for implementing brief interventions focused on reducing problem cannabis and alcohol use. Increased efforts and resources should be allocated for substance use prevention and treatment in PC settings.

## NC-P01 Brief intervention development for cancer patients who smoke: a review of the dialectical behavior therapy—skills training literature

### Marcia H. McCall^1^, Charlotte T. Boyd^1^, Nicole D. Kerr^2^

#### ^1^Wake Forest University School of Medicine, Winston-Salem, NC, USA; ^2^Wake Forest University, Winston-Salem, NC, USA

##### **Correspondence:** Marcia H. McCall (mmccall@wakehealth.edu)

*Addiction Science & Clinical Practice* 2023, **19(Suppl 1):**NC-P01

**Background:** Cancer patients who continue to smoke cigarettes after cancer diagnoses are at increased risk of death, treatment complications, and secondary tumors. However, the standard brief intervention for tobacco, behavioral counseling and medication, has not been effective for cancer patients, as it fails to treat their unique psychological barriers to addressing smoking such as stress and anxiety from having cancer, depression, and fatalistic beliefs. A promising alternative is Dialectical Behavior Therapy Skills Training (DBT-ST), a psychotherapeutic approach that helps people cope with negative emotions and stress so they can choose useful rather than destructive behaviors. DBT-ST brief interventions have been studied for alcohol and drug disorders, psychosocial stress of cancer diagnoses, severely anxious people, and underserved patient populations. A comprehensive review of this literature was unavailable but is necessary to establish feasibility and structure for a DBT-ST brief intervention for cancer patients.

**Materials and methods:** We conducted a literature review to locate studies of DBT-ST brief interventions of 20 sessions or less using keywords Dialectical Behavior Therapy, DBT, cancer, substance use, addiction, tobacco, and smoking. Databases searched included PubMed, PsycINFO, CINAHL, and the Cochrane Library. We compiled a list of studies meeting our criteria, then searched reference lists to find additional publications.

**Results:** Our review located 18 publications. Populations included people with drug use disorders, alcohol use disorders, various cancers, and other mental health conditions such as depression, anxiety, and suicidal ideation. DBT-ST brief interventions ranged from eight to 20 sessions. Consistently, patients found DBT-ST acceptable, with high completion rates versus control groups. Most studies demonstrated reductions in negative psychological symptoms and in substance use disorders, and/or improved quality of life.

**Conclusions:** DBT-ST is an acceptable, feasible, and effective brief intervention for populations and conditions similar to cancer patients who smoke, thus DBT-ST intervention development for this population is warranted.

## NC-P02 Using the Practical, Robust Implementation and Sustainability Model (PRISM) to evaluate implementations of tobacco cessation interventions in primary care clinics: a mixed methods systematic review

### Marcia H. McCall^1^, Benjamin D. Smart^2^, Sree V. Reddy^3^

#### ^1^Wake Forest University School of Medicine, Winston-Salem, NC, USA; ^2^Karolinska Institutet, Solna, Sweden; ^3^University of California San Diego Health, San Diego, CA, USA

##### **Correspondence:** Marcia H. McCall (mmccall@wakehealth.edu)

*Addiction Science & Clinical Practice* 2023, **19(Suppl 1):**NC-P02

**Background:** The health risks of tobacco use are well-established, and the benefits of quitting tobacco are clear, even after long-term use. However, of those attempting to quit, fewer than one third accessed evidence-based cessation treatment, and fewer than one in 10 were successful at quitting. Primary health care locations are opportunistic settings for delivering brief tobacco use intervention and referral to treatment. Most primary care providers routinely inquire about tobacco use, assess willingness, and advise patients to quit, but assistance and follow-up occur at fewer than one-third of visits. Providers cite lack of time, training, reimbursement, and awareness of treatment options. Implementation science offers frameworks and strategies to facilitate uptake of evidence-based tobacco use interventions in primary care practices. However, a systematic review of implementation studies to inform current practice and guide future research has not been done.

**Materials and methods:** We included multi-site studies in primary care settings with two or more implementation strategies. Using terms for primary care, tobacco use, and implementation science, we searched PubMed, Embase, CINAHL, PsycINFO, and the Cochrane Library, references lists of included articles, and gray literature. We evaluated our results with the Practical, Robust Implementation and Sustainability Model implementation framework.

**Results:** Our review yielded 38 publications, consisting of 24 randomized controlled trials, 11 non-randomized trials, and three other studies. The most common implementation strategies were leadership and community engagement, provider and staff training, feedback reports, provision of clinic and patient materials, medical record changes, role changes, program facilitation and financial incentives. Only six studies incorporated an implementation model. Across studies, implementation outcomes of reach, effectiveness, and adoption of tobacco intervention were achieved, but sustainability of programs was rarely considered.

**Conclusions:** Implementation studies employ multiple strategies and have demonstrated effectiveness for short-term outcomes, but future research should incorporate established implementation models and emphasize change sustainability.

## NC-P04 Perception of family support of alcohol and other drug users in treatment with brief intervention

### Rosa Peixoto^1^, Angela Abreu^1,2^, Marcia Peixoto^2^, Louise Paixão^1,2^, Larissa Mattos^1^, Lívia Falcão^1^, Thaina Silva^1^, Juliana Serpa^1^

#### ^1^Anna Nery School of Nursing, Federal University of Rio de Janeiro, Rio de Janeiro, RJ, Brazil; ^2^Department of Public Health, Federal University of Rio de Janeiro, Rio de Janeiro, RJ, Brazil

##### **Correspondence:** Rosa Peixoto (rosapeixoto.psi@gmail.com)

*Addiction Science & Clinical Practice* 2023, **19(Suppl 1):**NC-P04

**Background:** 284 million people aged 15–64 years (mostly men) have used drugs at least once in 2020 (UNODC, 2022). Brief Intervention has proven effective in addressing drug-related problems and can be enhanced with the presence of social support, especially family support.

**Objective:** To evaluate the family support perceived by patients that use alcohol and other drugs that are in treatment with Brief Intervention.

**Methods:** Cross-sectional study, carried out in a Medium Complexity Care Unit of a University Hospital, Rio de Janeiro/Brazil, with psychoactive substance users in treatment with Brief Intervention. It was approved by the Research Ethics Committee of EEAN/UFRJ. The sample was composed of 35 patients. The data related to the socio-demographic profile and to the Family Support Perception Inventory (IPSF) were collected through interviews with the patients. The ASSIST data were collected by consulting the participants’ medical records. Descriptive statistics with simple frequencies and percentages were used for data analysis, performed in SPSS Version 21.

**Results:** It was observed a predominance of males (94.3%), in the age group of 40 to 57 years (74.3%), with an average of 46.5 years old, incomplete elementary education (34.3%), majority black or brown (74.3%), no marital partnership (68.6%). The predominant using substances were cocaine (54.3%) and alcohol (45.7%). The average score of the first ASSIST for alcohol was 22.9 and for cocaine, 17.67. And 65.7% of the participants claimed to receive no support from family members for treatment. Family support was perceived as low by 32.1%, medium–low by 22.9%, medium–high by 17.1%, and high by 22.9%.

**Conclusion:** It was noticed a clientele with a perception of low family support. Study in progress. Further analyses will be carried out in the search for association of family support with the results of the Brief Intervention, to be presented at the INEBRIA Conference.

## NC-P06 Significant clinical change in the brief intervention for adolescent alcohol abusers

### Eunice Vargas-Contreras^1^, Ana Lucía Jiménez-Pérez^1^, Norma Yadira Estrada-Vergara^1^, Ilse Abigail Arreola-Sánchez^1^, Kalina Isela Martínez-Martínez^2^

#### ^1^Autonomous University of Baja California. Faculty of Administrative and Social Science, Ensenada, Baja California, Mexico; ^2^Autonomous University of Aguascalientes, Department of Social Science and Humanities, Aguascalientes, Mexico

##### **Correspondence:** Eunice Vargas-Contreras (eunice.vargas@uabc.edu.mx)

*Addiction Science & Clinical Practice* 2023, **19(Suppl 1):**NC-P06

**Background:** The studies about the efficacy of substance abuse treatmen, only consider the statistical significance of the change in the reduction of alcohol consumption, however, the clinical significance and the size of the effect of an intervention provides data from sample to be compared with a normal population, giving evidence of the degree to which the sample attain normal levels of behavior. The objective was to determine the clinical significance of the change and the size of the effect, generated by the Brief Intervention for Adolescents who initiate substance abuse (PIBA).

**Material and methods:** A pretest–posttest design was carried out, participated 100 adolescents between 14 and 18 years old. A retrospective baseline and self-report was applied to count the drinks per occasion and consumption days.

**Results:** Results show statistically significant differences in the amount of consumption per occasion, as well as in the frequency of consumption. Clinically significant change was found, and the alcohol consumption of the sample corresponded to that of the population without risk; the effect size was moderate.

**Conclusion:** PIBA is an effective intervention in the reduction of alcohol consumption, it is necessary to continue the research about another drugs consumption. It is important to analyze the findings from the implementation, after the program transfer process in clinical settings.

## NC-P08 Brief intervention in a primary care unit for problems related to the consumption of alcohol and other drugs as a service practice and policy

### Angela Abreu^1,2^, Rosa Peixoto^1^, Marcia Peixoto^2^, Louise Paixão^1,2^, Larissa Mattos^1^, Lívia Falcão^1^, Thaina Silva^1^, Juliana Serpa^1^

#### ^1^Anna Nery School of Nursing, Federal University of Rio de Janeiro, Rio de Janeiro, RJ, Brazil; ^2^Department of Public Health, Federal University of Rio de Janeiro, Rio de Janeiro, RJ, Brazil

##### **Correspondence:** Angela Abreu (angelamendesabreu@gmail.com)

*Addiction Science & Clinical Practice* 2023, **19(Suppl 1):**NC-P08

**Background:** UNODC/WHO, adds that only one in eight people with drug use disorders received professional help in 2019. The consequences of the shortage of these services were most felt in the poorest countries.

**Objective:** To describe the effectiveness of the Brief Intervention, in a Primary Care unit for patients who use alcohol and other drugs, as a service policy.

**Methods:** Brief Intervention Implementation Protocol in a Unit of a University Hospital in Rio de Janeiro, Brazil. Field of practice for undergraduate and graduate students. Real-world longitudinal study conducted from July 2021 to December 2022 with 158 patients, using the ASSIST questionnaire. Inclusion criteria, patients attended at the unit, who had undergone the consultation, using the Brief Intervention technique, the cutoff point being considered as adherence from 3 consecutive consultations. The study was approved by the Research Ethics Committee of the EEAN/UFRJ.

**Results:** Initially, all attended by the Social Service and referred to the nurses/professors consultation, on Tuesdays and Thursdays and with students. So far, we have preliminary data, with a sample of 28 patients analyzed. Male (78.6%), over 40 years of age (75.0%), complete primary education (39.3%), income less than one minimum wage (50.0%), religious (89.3%). Cocaine was the most used substance followed by alcohol, marijuana and tobacco. With 46.4% of patients ceasing use within 12 weeks. Between the first and second ASSIST, a decrease in the p < 0.001 score was observed and 92.9% of the patients showed a reduction in use.

**Conclusions:** Preliminary results. Noticed the effectiveness, after the implementation of the Brief Intervention technique in the reduction and cessation of substance use. Protocol, became the guiding principle for the practice and service policy at the Unit. Study is still in progress for presentation at INEBRIA.

## NC-P09 Barriers to enrolling monolingual Spanish-speaking primary care patients in a screening and brief intervention (SBI) to reduce risky drug use: lessons learned from the QUIT-Mobile study

### Leticia Cazares^1^, Amanda Sirisoma^1^, Cristina Batarse^1^, Cristina Hernandez^1^, Stephanie Sumstine^2^, Melvin W. Rico^1,4^, Dallas Swendeman^2^, Lillian Gelberg^1,3^

#### ^1^Department of Family Medicine, David Geffen School of Medicine at UCLA, Los Angeles, California, USA; ^2^Department of Psychiatry and Biobehavioral Sciences, David Geffen School of Medicine at UCLA, Los Angeles, California, USA; ^3^Department of Health Policy & Management, Fielding School of Public Health, UCLA, Los Angeles, California, USA; ^4^Charles R. Drew University of Medicine and Science, Los Angeles, California, United States

##### **Correspondence:** Cristina Batarse (cristinabatarse@gmail.com)

*Addiction Science & Clinical Practice* 2023, **19(Suppl 1):**NC-P09

**Background:** QUIT Using Drugs Intervention Trial Mobile Study (QUIT-M) is a Screening and Brief Intervention (SBI) effectiveness-implementation RCT to reduce moderate substance use among primary care patients of federally qualified health centers (FQHCs) in Los Angeles. This analysis details barriers in enrolling monolingual Spanish-speaking patients.

**Materials and methods:** Patients with a primary care appointment are sent a self-administered screener with the WHO ASSIST to determine eligibility. QUIT-M consists of a PCP brief advice, telephone health coaching and text message self-management support. Study materials are translated in Spanish and were reviewed to ensure cultural appropriateness.

**Results:** Of 9537 patients who received the screener from April 2022–April 2023, 3250 completed it (34% completion rate) with a higher rate of non-response among Latinx patients. Of 3250 screened, 1821 identified as Latinx (56%). Of 1821 Latinx patients who completed it, the common reason for screening ineligible was: “reporting no drug use in lifetime (n = 1350)”. Of 92 enrolled patients, 3 are monolingual Spanish speaking. Common themes identified through patient interactions regarding lack of screener completion among Spanish speakers included: (1) distrust and discomfort about discussing drug use; (2) patients becoming offended due to feeling disrespected because they don’t want to be perceived as users given the stigma; (3) reporting not knowing how to use smartphones beyond making phone calls. Monolingual Spanish speaking patients preferred in-person interaction with the study team in clinics to build trust.

**Conclusions:** Monolingual Spanish speaking patients are more likely to report no drug use in their lifetime, which led them to screen ineligible. Distrust and discomfort with screening may be due to fear of reporting sensitive drug use or of drug-related questions being tied to medical record and legal consequences. It may be beneficial to develop a culturally sensitive screening process that includes bilingual and bicultural staff that focus on destigmatizing reporting substance use.

## NC-P11 A qualitative exploration of barriers and facilitators of alcohol screening brief interventions (ASBI) undertaken by paramedics in the North East of England

### Christopher Moat^1^, Jennifer Ferguson^1^, Newbury-Birch^1^, Katie Haighton^2^

#### ^1^Teesside University, Middlesbrough, England, UK; ^2^Northumbria University, Newcastle, England, UK

##### **Correspondence:** Christopher Moat (c.moat@tees.ac.uk)

*Addiction Science & Clinical Practice* 2023, **19(Suppl 1):**NC-P11

**Background:** In the UK NHS Ambulance service staff do not currently undertake any ASBI activities. This research study aims to explore the feasibility of carrying out ASBI’s by UK Paramedics.

**Methods:** Qualitative study of 16 student paramedics in the North East of England. Participants were enrolled upon year 2 of an approved BSc program of study. Prior to data collection a 30-min education session discussing alcohol-related presentations and ASBI’s was provided. Participants demographic data, collected via an anonymous survey. Participants participated in small focus groups, four weeks post education session. Focus groups were repeated, following additional clinical exposure.

**Findings:** Similarities emerged across all focus groups. Participants felt helpless, unable to assist service user’s, recognising a lack of knowledge and understanding. Some participants felt this patient group overshadowed more acute presentations but accepted that these patients required help and the ambulance service were uniquely positioned to assist. All participants agreed they did not truly understand screening, its appropriateness and questioned the availability of referral pathways. Repeat focus group’s suggested participants did feel this study had improved their practice and identified the need for further training and referral pathways, all agreed screening of this patient group was an additional assessment a suitable for Paramedics.

**Conclusion:** To improve the experiences of service users and Paramedics healthcare providers should develop further training for staff and referral pathways require further development to improve patient contact experiences and ensure appropriate referrals can be made.

## NC-P12 Evaluating brief interventions using coproduction methods alongside a local authority in the North East of England

### Natalie Connor^1^, Andy Divers^1^, Kirsty Wilkinson^2^, Dorothy Newbury-Birch^1^

#### ^1^Teesside University, Middlesbrough, England, UK; ^2^Durham County Council, County Durham, England, UK

##### **Correspondence:** Natalie Connor (n.connor@tees.ac.uk)

*Addiction Science & Clinical Practice* 2023, **19(Suppl 1):**NC-P12

**Background:** For ten years, academic researchers from Teesside University have worked collaboratively with public health practitioners from Durham County Council on a series of coproduced evaluations. A researcher-in-residence model has been adopted with a working pattern whereby researchers hold an honorary position at the local authority and vice versa. This has allowed for an understanding of the different processes and cultures to develop; and provided opportunities to develop evaluation projects together. Several evaluations have explored brief interventions or the potential for brief interventions as part of the service.

**Methods:** Evaluations have involved an academic researcher and public health practitioners working together on all aspects of evaluations, from protocol design to dissemination of results. The methods adopted for each of the evaluations were unique depending on the nature of the service, however most were either a process or outcome evaluation. In total, 21 public health evaluations have taken place involving five academic researchers, and 24 public health portfolio leads. One of the current evaluations, an evaluation exploring the County Durham Mental Health Employment Practitioner Service, has the opportunity to include a brief intervention.

**Findings:** Five out of the 21 evaluations have explored brief interventions. They have covered the areas of mental health support, domestic violence, employment seeking, smoking cessation, alcohol use, cancer support, loneliness and isolation, debt management, falls in the home and weight management. As part of evaluation findings, opportunities for brief interventions have been identified, and recommendations put into place as to how they can be implemented. Brief interventions have also aligned with the ‘Making Every Contact Count’ approach to behaviour change.

**Conclusion:** Local authorities are well placed to deliver brief interventions, and coproduced evaluations with an academic partner can ensure that best practice is highlighted, and recommendations for service improvements are supported by an evidence-base.

## NC-P13 “If in the First Act, you hang a gun upon the wall, by the Third Act, you must use it” (Chekhov) exploring the complex ethical issues around research into brief interventions

### Andrew Divers, Maddison Din, Dorothy Newbury-Birch

#### Teesside University, Middlesbrough, UK

##### **Correspondence:** Andrew Divers (a.divers@tees.ac.uk)

*Addiction Science & Clinical Practice* 2023, **19(Suppl 1):**NC-P13

The factors contributing to alcohol and substance use are complex, as are the individuals who are involved in researching such phenomena. It is clear that when conducting research with potentially vulnerable individuals, particular care should be taken by researchers to properly fulfil their ethical duties to participants. What is less clear, however, is what this may look like—and more importantly—what this ought to look like. This presentation will briefly explore some of the key questions that researchers within the field of brief interventions should be asking themselves before they design and embark on studies, and will also outline how to ensure their research maintains the highest ethical standards when interacting with participants, encompassing the limits of informed consent (O’Neill, 2003) data solidarity (Prainsack et al., 2022) and how to ensure we avoid prefatory rubrics (Flew, 1954) and simply “testing out our new sword” (Midgley, 2003).

## NC-P14 Comparison of implementing SBIRT in the ED vs the trauma floor in a trauma one hospital

### Janice Williams

#### Department of Emergency Medicine and Trauma Services, Atrium Health, Charlotte, NC, USA

##### **Correspondence:** Janice Williams (janice.williams@atriumhealth.org)

*Addiction Science & Clinical Practice* 2023, **19(Suppl 1):**NC-P14

**Background:** Substance use is under reported when present in incidents resulting in severe injury or fatal outcomes. Currently 14% in the local community of service for impaired road crashes (NC vision zero analytics). SBIRT is a known evidenced based intervention recently explored in a variety of settings with few tools from boots on the ground implementors collecting variables to help those implementing in specific settings to help tailor the program.

**Methods:** A trauma one center over the past two decades has implemented SBIRT in the same healthcare system in a community in different departments and collected barriers, facilitators, staff needs, patient profiles, and outcomes.

**Results:** Emergency Room implementation is successful when specific staff, timing, patient profile, and treatment access tuning is attained to and is to date the most successful cost savings and satisfaction outcome setting with $700,000 saving in a year compared to traditional case management support. Trauma inpatients are successful more with harm planning and counseling sessions since less than 1% qualify or accept a treatment referral and present with a lower readiness to change indicator. In both settings, provider and patient interest is high in adoption with help of translation support.

**Conclusion:** When SBIRT can be adopted with tailored support to patients, setting, and providers, it can be successful in adoption across a variety of settings with positive outcomes. But more intervention level data needs to be collected related to implementation science for widespread use without dedicated staff.

